# Chemotherapy-induced gastrointestinal toxicity is associated with changes in serum and urine metabolome and fecal microbiota in male Sprague–Dawley rats

**DOI:** 10.1007/s00280-017-3364-z

**Published:** 2017-06-23

**Authors:** Richard A. Forsgård, Vannina G. Marrachelli, Katri Korpela, Rafael Frias, Maria Carmen Collado, Riitta Korpela, Daniel Monleon, Thomas Spillmann, Pia Österlund

**Affiliations:** 1 Pharmacology, Faculty of Medicine, University of Helsinki, P.O. Box 63, 00014 Helsinki, Finland; 2 Metabolomics and Molecular Imaging Lab, Health Research Institute INCLIVA, Valencia, Spain; 3 Immunobiology Research Program, Department of Bacteriology and Immunology, University of Helsinki, Helsinki, Finland; 4 Central Animal Laboratory, University of Turku, Turku, Finland; 5 Comparative Medicine, Karolinska Institutet, Stockholm, Sweden; 6 Institute of Agrochemistry and Food Technology, National Research Council (IATA-CSIC), Valencia, Spain; 7 Department of Equine and Small Animal Medicine, Faculty of Veterinary Medicine, University of Helsinki, Helsinki, Finland; 8 Department of Oncology, University of Helsinki and Helsinki University Hospital, Helsinki, Finland; 9 Department of Oncology, Tampere University Hospital, Tampere, Finland

**Keywords:** Chemotherapy, Metabolomics, Microbiota, 5-FU, Oxaliplatin, Irinotecan

## Abstract

**Purpose:**

Chemotherapy-induced gastrointestinal toxicity (CIGT) is a complex process that involves multiple pathophysiological mechanisms. We have previously shown that commonly used chemotherapeutics 5-fluorouracil, oxaliplatin, and irinotecan damage the intestinal mucosa and increase intestinal permeability to iohexol. We hypothesized that CIGT is associated with alterations in fecal microbiota and metabolome. Our aim was to characterize these changes and examine how they relate to the severity of CIGT.

**Methods:**

A total of 48 male Sprague–Dawley rats were injected intraperitoneally either with 5-fluorouracil (150 mg/kg), oxaliplatin (15 mg/kg), or irinotecan (200 mg/kg). Body weight change was measured daily after drug administration and the animals were euthanized after 72 h. Blood, urine, and fecal samples were collected at baseline and at the end of the experiment. The changes in the composition of fecal microbiota were analyzed with 16S rRNA gene sequencing. Metabolic changes in serum and urine metabolome were measured with 1 mm proton nuclear magnetic resonance (^1^H-NMR).

**Results:**

Irinotecan increased the relative abundance of Fusobacteria and Proteobacteria, while 5-FU and oxaliplatin caused only minor changes in the composition of fecal microbiota. All chemotherapeutics increased the levels of serum fatty acids and N(CH_3_)_3_ moieties and decreased the levels of Krebs cycle metabolites and free amino acids.

**Conclusions:**

Chemotherapeutic drugs, 5-fluorouracil, oxaliplatin, and irinotecan, induce several microbial and metabolic changes which may play a role in the pathophysiology of CIGT. The observed changes in intestinal permeability, fecal microbiota, and metabolome suggest the activation of inflammatory processes.

**Electronic supplementary material:**

The online version of this article (doi:10.1007/s00280-017-3364-z) contains supplementary material, which is available to authorized users.

## Introduction

Chemotherapy-induced gastrointestinal toxicity (CIGT) is a common complication of cancer treatment that can lead to a variety of symptoms [1]. Gastrointestinal symptoms such as diarrhea, weight loss, pain, and infections are serious adverse events that may significantly harm the patients’ prognosis due to dose reductions and treatment interruptions [1, 2]. Thus, a better understanding of the pathophysiological mechanisms of CIGT could offer tools for managing these complications better and possibly improving treatment outcomes.

The pathophysiology of CIGT is complex and most likely involves multiple different processes. Studies have shown that chemotherapeutic drugs increase intestinal permeability, induce the generation of reactive oxygen species (ROS) and pro-inflammatory cytokines, and modulate gut microbiota [3, 4]. Gut microbiota is an important regulator of intestinal homeostasis and alterations in microbial composition have been associated with multiple inflammatory diseases. Recent advances have also led to the hypothesis that microbiota plays a role in the development of CIGT [5] and in some cases even predict chemotherapy-related toxicities [6]. Hence, characterizing the chemotherapy-induced changes in microbiota composition may reveal new insights into the development and management of CIGT.

Metabolomics offers a system-level approach to study the metabolic state of a biological system and alterations in the system’s metabolome can reveal new insights about disease pathophysiology. Researchers have applied metabolomics to finding novel biomarkers for a variety of diseases such as inflammatory bowel diseases (IBDs) and cancer [7–9]. In addition to diseases, studies have shown that metabolic profiling can assess and predict drug response and toxicity [10–13]. Thus, the so-called pharmaco-metabolomics has emerged as a potentially useful tool in personalized healthcare [10]. However, data regarding the pharmaco-metabolic profile of chemotherapeutic drugs remain limited [14]. Finding clinically relevant biomarkers of CIGT would help clinicians to optimize treatment efficacy and reduce the severity and complications of adverse events.

We have previously shown that 5-fluorouracil, oxaliplatin, and irinotecan increase intestinal permeability to iohexol, and the increase in permeability also correlates with the severity of the clinical signs of CIGT [15]. In this study, we hypothesized that the gastrointestinal toxicity induced by 5-fluorouracil, oxaliplatin, and irinotecan could be associated with changes in fecal microbiota and the metabolome in serum and urine. Our aim was to characterize these changes and examine how they relate to the severity of CIGT and to the observed increase in intestinal permeability to iohexol.

## Materials and methods

### Ethical statement

The experiment using animals was approved by the National Animal Experiment Board in Finland (ESAVI/114/04.10.07/2015).

### Animals

All animals included in this study were healthy and naïve (never exposed to any drug or procedure before) male Hsd:Sprague–Dawley^®^™ SD^®^™ (SD) rats obtained from a reputable breeding company (Envigo, Udine, Italy) at the age of 5 weeks. The rats were housed in a specific pathogen-free rodent facility in open stainless steel cages (59.5 × 38.0 × 20 cm) with solid bottoms and Aspen chips as bedding (Tapvei, Harjumaa, Estonia). The husbandry conditions in which the animals were housed included artificial lighting of 12-h light/dark cycle, an average room temperature of 22 ± 2 °C, and a relative humidity of 55 ± 15%. The animals had free access to rat chow (2018 Teklad Global 18% Protein Rodent Diet, Harlan Laboratories, Madison, WI, USA) and tap water throughout the study. The rats were allowed to acclimatize for 18 days before the start of the experimental protocol.

### Experimental protocol

**Fig. 1:**
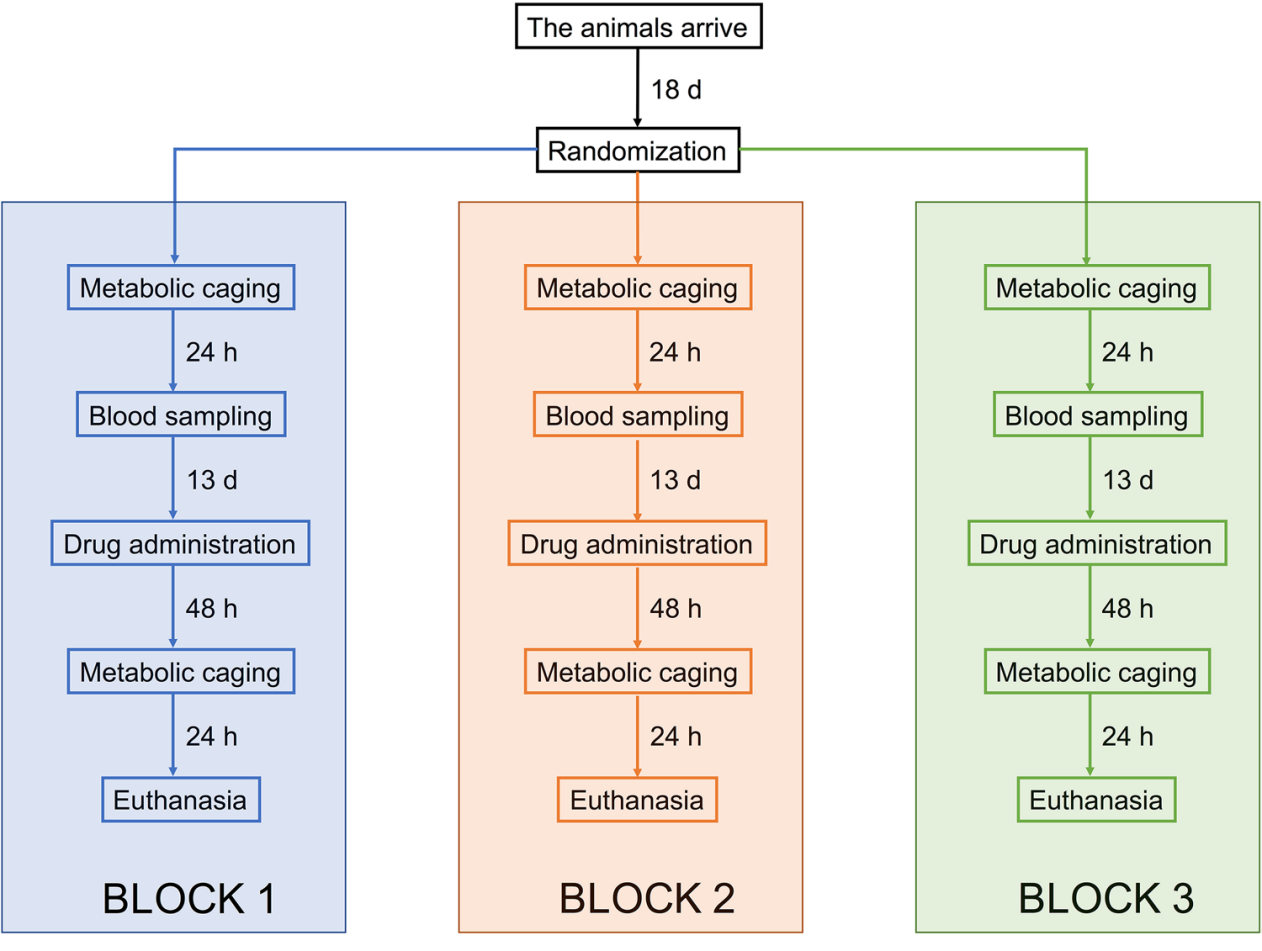
Flow chart illustrating the “Experimental protocol”. The rats were allowed to acclimatize for 18 days before they were randomized into four treatment groups (control, 5-fluorouracil, oxaliplatin, and irinotecan) and the experimental protocol was started by collecting baseline samples (metabolic caging and blood sampling). Following a 13-day recuperation period, the rats were injected intraperitoneally with either 0.9% saline, 150-mg/kg of 5-fluorouracil, 15-mg/kg oxaliplatin, or 200-mg/kg irinotecan. After 48 h, the rats were once again placed in metabolic cages for 24 h after which they were euthanized. The experimental protocol was conducted in three blocks where each block consisted of 16 animals (four animals from each group). Parallel procedures in* each block* were conducted with 1-day intervals

At the start of the experimental protocol, the rats were 8 weeks old and the average body weight was 283 ± 16 g. The experimental protocol has been previously described in Forsgård et al. [15]. Briefly, a total of 48 rats were randomized into four experimental groups: control, 5-fluorouracil, oxaliplatin, and irinotecan (*n* = 12 in each group). The experimenter was aware of group identities throughout the experimental protocol. Baseline measurements were started by weighing the animals and placing them in individual metabolic cages for 24 h after which urine and feces were collected and stored in −80 °C for later analysis. Baseline intestinal permeability was also assessed at this point (“Measurement of intestinal permeability”). After metabolic caging, a 1-ml blood sample was taken from the tail lateral vein under isoflurane (Vetflurane 1000 mg/g, Virbac, Suffolk, UK) anesthesia. After a 13-day recuperation period, the animals were administered with the chemotherapeutic drugs (“Drug dosing”) after which the animals were weighted daily. After 48 h, the rats were again placed individually in the metabolic cages for 24 h for urine and feces collection. Intestinal permeability was again measured at this point. After metabolic caging, all the animals were humanely sacrificed by inducing unconsciousness with isoflurane and subsequent exsanguination by cardiac puncture and by severing of the aorta. The experimental protocol was executed in three blocks where each block consisted of 16 animals (4 from each group). The experimental protocol is illustrated in Fig. 1.

### Drug dosing

The rats were injected intraperitoneally with either 0.9% saline (control), 150-mg/kg 5-fluorouracil (Accord Healthcare, Middlesex, UK), 15-mg/kg oxaliplatin (Hospira UK, Warwickshire, UK), or 200-mg/kg irinotecan (Hospira UK, Warwickshire, UK). To reduce the irinotecan-induced cholinergic reaction, a subcutaneous injection of 0.01-mg/kg atropine (Leiras, Espoo, Finland) was given immediately prior to irinotecan administration. All injections were administered under isoflurane anesthesia.

### Blood sampling

The blood samples were collected in serum separation tubes (VenoSafe™ Clot Act. (Z), Terumo Europe, Leuven, Belgium) and centrifuged at 1500*g* for 10 min at 4 °C. The separated serum was collected and stored in −80 °C for later analysis.

### Measurement of intestinal permeability

Intestinal permeability was measured by administering all rats with 1 ml of 647-mg/ml iohexol solution (Omnipaque 300™, 647-mg iohexol/ml, GE Healthcare, Oslo, Norway) by oral gavage immediately prior to placing them in individual metabolic cages for 24 h. The urinary iohexol concentration was measured by enzyme-linked immunosorbent assay (ELISA) per the manufacturer’s instructions (BioPAL Inc., Worcester, MA, USA). The percentage of excreted iohexol was calculated using the following equation:

$${\text{Iohexol (}}\% ) = {\text{amount of iohexol excreted in urine after 24}}\;{\text{h (mg)}}/{\text{amount of administered iohexol (mg)}}\, \times \, 100.$$

### Fecal microbiota analysis

Microbial DNA from rat fecal samples was extracted with QIAamp DNA MinikIt (Qiagen, Doncaster, UK). Briefly, the fecal pellets were first homogenized in lysis buffer and nucleic acids were precipitated with isopropanol. The nucleic acid pellet was dissolved in 10-mM Tris—1-mM EDTA buffer and the DNA was purified according to the kit manufacturer’s instructions. The DNA concentration of the samples was determined using Quant-iT™ PicoGreen^®^ Assay (Invitrogen, Eugene, OR, USA) and the samples were subsequently diluted to a concentration of 1 ng/μl.

The bacterial composition in the fecal samples was analyzed using 16S rRNA gene sequencing on the Illumina HiSeq 2500 platform. PCR amplification was done in two steps and DNA libraries were constructed using the Illumina Nextera kit. Paired-end sequencing of 200-nt-reads was performed. In total, the sequencing produced 9698,319 reads, on average 115,423 per sample. The sequences were processed using the program R [16] and the package mare [17] based on a previously validated protocol [17]. Only the forward read was used, truncated to 150 nt to remove the low-quality end of the reads. Quality filtering based on prevalence of unique reads was conducted: unique reads representing less than 0.01% of all reads were excluded as potentially erroneous. Chimera filtering was done using UCHIME in the denovo mode [18]. OTU clustering was not done; instead, the reads were taxonomically annotated and then summarized at different taxonomic levels. Taxonomic annotation of the reads was performed using UPARSE [19] using 0.5 as the confidence cutoff and the RDP as the reference database.

## 1-mm proton nuclear magnetic resonance (^1^H-NMR) analysis of serum and urine

For ^1^H-NMR analysis, 20 µl of serum were mixed with 2.5 µl of sodium-3′-trimethylsilylpropionate-2,2,3,3-d4 (TSP, 2.5 mM) in deuterium oxide (D_2_O). For urine samples, 2 µl of a phosphate buffer solution (0.06 M Na_2_HPO_4_/0.04-M NaH_2_PO_4_, pH 7) and TSP 2.5-mM was added to overcome the pH variation problem. A total of 20 µl of the mixture of each sample were then transferred into a 1-mm high-quality NMR tube individually. All spectra were acquired using a standard one-dimensional pulse sequence with water suppression (Bruker Avance 600 spectrometer operating at 600.13 MHz with a 1-mm 1H/13C/15N TXI probe). Nominal temperature of the sample was kept at 310 K. A single-pulse presaturation experiment was acquired in all the samples. Number of transients was 256 collected into 65 K data points for all the experiments. Water signal was saturated with a weak irradiation during the recycle delay. Before Fourier transformation, the free induction decay was multiplied by a 0.3-Hz exponential line broadening. All spectra were processed using MestReNova 8.1 (Mestrelab Research S.L., Spain) and transferred to MATLAB (MathWorks) using in-house scripts for data analysis. The chemical shift region including resonances between 0.50 and 4.30 ppm (the aliphatic region) and 5.20–9.5 ppm (the aromatic region) was investigated. The spectra were binned into 0.01-ppm buckets and mean centered for multivariate analysis and normalized to total aliphatic spectral area to eliminate differences in metabolite total concentration. Every compound was identified using literature data and the commercial resonances database Chenomx (Chenomx NMR 7.6). Peaks were assigned considering the chemical shift relative to TSP and the spin–spin coupling patterns. TSP was used as a standard compound in peak assigning, because its signal is always in the same position and does not overlap with other signals. To minimize noise, the spectra were reduced to 66 and 111 regions for serum and urine, respectively, based on its metabolites enrichment. Peak area integration was used to calculate the relative contributions. Signals belonging to selected regions were quantified using semi-automated in-house MATLAB (MathWorks) integration and peak-fitting routines. Quantification was assessed for spectral regions containing contributions of a single or at most two metabolites. For the remaining metabolites, quantification was compromised due to low signals and/or overlapping.

### Data analysis

In the fecal microbiota analysis, mare package [17] was used to create the taxonomic tables, count the relative abundances of each taxon, and calculate the inverse Simpson diversity index. The differences in the relative abundances of bacterial taxa at baseline and at the end of experiment were analyzed by the Kruskal–Wallis test, and if global *p* < 0.05, the differences between groups were tested with post hoc Dunn–Bonferroni test with Bonferroni correction. However, the change test script with false discovery rate correction (FDR) in the mare package was used to identify treatment-induced significant changes from baseline in the relative abundance of bacterial taxa between the groups. Differences in inverse Simpson index were first analyzed with one-way ANOVA followed Bonferroni post hoc test. All microbial changes were deemed significant when *p* < 0.05. The differences in relative metabolite concentrations between the control group and the treatment groups were calculated with multivariate paired analysis (SPSS Statistics 22.0, IBM, Armonk, NY, USA). The *p* values were recalculated by Bonferroni correction and *p* values <0.05 were deemed significant. The metabolomic statistical analysis was performed using in-house MATLAB scripts and the PLS Toolbox (Eigenvector Research) statistical multivariate analysis library. Principle component analysis (PCA) and partial least squares-discriminant analysis (PLS-DA) were carried out to visualize the metabolic alterations between groups after mean centering and unit variance scaling. The main advantage of PCA and PLS-DA models is that the main sources of variability in the data are modeled by new variables that explain most of the observed variance in the data. Consequently, the associated scores and loadings allow the visualization and understanding of different patterns and relations in the data. The variable importance in the projection (VIP) values of all the peaks from the tenfold cross-validated PLS-DA model was taken as a coefficient for peak selection. A variable with a VIP Score close to or greater than one was considered relevant for group discrimination in each model. Correlations between the relative abundances of bacterial phyla, inverse Simpson index, metabolite levels, intestinal permeability, and body weight change were calculated as Pearson’s correlations and visualized using the corrplot package [20].

## Results

### Drug response

Detailed description of the CIGT and changes in intestinal permeability to the drugs has previously been published in Forsgård et al. [15]. Briefly, after administration of the chemotherapeutics, all treatment groups experienced significant body weight loss and an increase in intestinal permeability to iohexol compared to the control group (*p* < 0.001) (Table 1).

**Table 1:** Intestinal permeability to iohexol (IP) and body weight change (BWC) in different treatment groups 72 h after drug administration

	Group				
	Control	5-Fluorouracil	Oxaliplatin	Irinotecan	Kruskal–Wallis
IP (%)	0.47 ± 0.18^b^	1.6 ± 1.5^a,b^	2.6 ± 1.5^a,b^	8.1 ± 8.9^b^	*p* < 0.001
BWC (%)	0.88 ± 2.6^b^	−6.6 ± 2.3^b^	−11.6 ± 3.4^b^	−16.0 ± 3.5^b^	*p* < 0.001

Results are expressed as medians ± interquartile ranges

^a^Statistically significant difference between groups (*p* < 0.05)

^b^Statistically significant difference between groups (*p* < 0.001)

### Fecal microbiota analysis

**Fig. 2:**
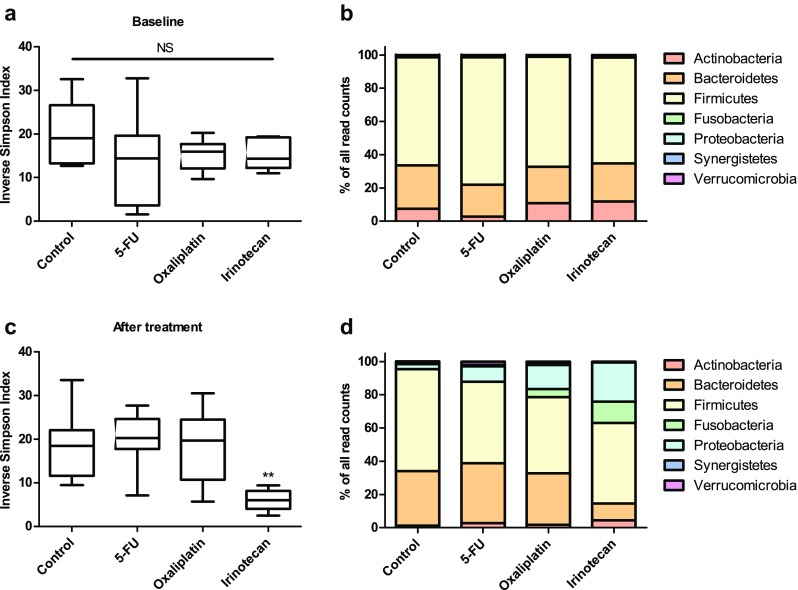
Analysis of fecal microbiota with 16S rRNA gene sequencing revealed that there were no differences in microbial diversity (inverse Simpson index) between groups at baseline (**a**). At the phylum level, the microbial composition was similar in all groups except in the 5-fluorouracil group which exhibited elevated relative abundance of Firmicutes and decreased relative abundance of Actinobacteria (**b**). At the end of the experiment, irinotecan had induced a significant decrease in microbial diversity compared to the other groups (**c**). The administered chemotherapeutics caused significant changes in the composition of fecal microbiota (**d**). Irinotecan treatment increased the relative abundances of Fusobacteria and Proteobacteria as well as decreased the relative abundances of Actinobacteria, Bacteroidetes, and Synergistetes. In addition, 5-fluorouracil administration increased the relative abundance of Verrucomicrobia. *Box plots* (**a**, **c**) show median with* upper* and* lower* quartiles. *Bar graphs* (**b**, **d**) show the mean relative abundance of each microbial phyla in each treatment group. *Whiskers* show minimum and maximum. (**p < 0.01)

**Fig. 3:**
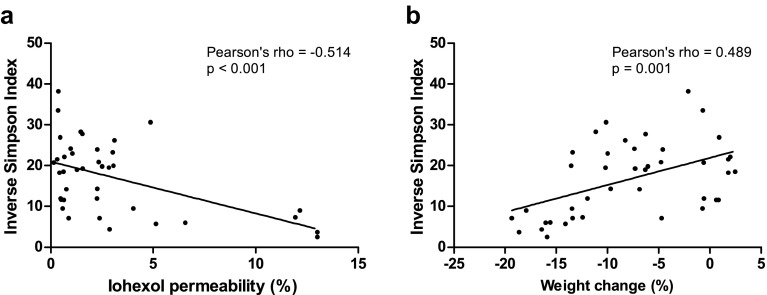
Pearson correlations between fecal microbiota diversity (inverse Simpson index) at the end of the experiment and intestinal permeability to iohexol (**a**), and fecal microbiota diversity at the end of the experiment and body weight change (%) (**b**). Fecal microbiota diversity correlated inversely with iohexol permeability (Pearson’s *ρ* = –0.514, *p* < 0.001) and positively with body weight change (Pearson’s *ρ* = 0.489, *p* = 0.001)

At baseline, there were no statistical differences in fecal microbiota diversity (inverse Simpson index) between control and treatment groups (Fig. 2a). However, at the end of the experiment, the Irinotecan group showed a significantly (*p* < 0.01) reduced fecal microbiota diversity compared to all other groups (Fig. 2c). Fecal microbiota diversity also correlated inversely with iohexol permeability (*p* = 0.001) and positively with body weight loss (*p* = 0.002) (Fig. 3) at the end of the experiment. At the phylum level, at baseline, rats in the 5-FU group had significantly lower levels of Actinobacteria compared to those observed in the oxaliplatin and irinotecan groups (*p* < 0.05 and *p* < 0.01, respectively). The 5-FU group also had significantly higher levels of Firmicutes at baseline than the control and Irinotecan group (*p* < 0.05 and *p* < 0.01, respectively) (Fig. 2b; Table 2). At the end of the experiment, the irinotecan group exhibited significantly decreased relative abundances of Actinobacteria (*p* < 0.05), Bacteroidetes (*p* < 0.001), and Synergistetes (*p* < 0.001) compared to the control group (Fig. 2d; Table 2). Irinotecan also induced a significant increase in the relative abundances of Fusobacteria (*p* < 0.001) and Proteobacteria (*p* < 0.01) compared to the control group. Treatment with 5-FU caused a significant (*p* < 0.01) rise in the relative abundance of Verrucomicrobia compared to the control group. The Oxaliplatin group exhibited significantly (*p* < 0.05) higher relative abundance of Proteobacteria than the control group at the end of experiment, but the change from baseline was not statistically significant. All phylum level changes are summarized in Table 2. At the genus level, we found 24 taxa with a statistically significant change from baseline compared to the control group (Table 3). All genus level changes in relative abundances are summarized in Online Resource 1.

**Table 2:** Relative abundances (% of all reads) of each bacterial phylum at baseline (*t*1) and at the end of the experiment (*t*2)

Phylum	Control			5-Fluorouracil			Oxaliplatin			Irinotecan		
	*t*1 (%)	*t*2 (%)	Δ (%)	*t*1	*t*2 (%)	Δ (%)	*t*1	*t*2 (%)	Δ (%)	*t*1	*t*2	Δ
Actinobacteria	7.6 ± 7.3	1.3 ± 0.6	−5.9 ± 6.0^a^	2.7 ± 1.8%^a,b^	2.7 ± 3.8	0.01 ± 3.3	10.8 ± 10.4%^a^	1.7 ± 2.2	−9.4 ± 10.7	11.9 ± 7.4%^b^	2.7 ± 8.0	−8.0 ± 9.5^a^
Bacteroidetes	26.1 ± 14.1	29.8 ± 16.5^b^	4.1 ± 23.4^c^	19.2 ± 14.2%	37.2 ± 8.2^c^	19.7 ± 19.1^c^	22.0 ± 8.9%	30.0 ± 12.2^a^	10.4 ± 18.0^c^	22.8 ± 10.8%	9.5 ± 12.4%^a,b,c^	−16.4 ± 22.0^c,c,c^
Firmicutes	65.1 ± 8.8^a^	62.8 ± 16.0	−1.9 ± 28.6	76.7 ± 12.5%^a,b^	51.3 ± 16.2	−27.8 ± 18.8	66.1 ± 8.1%	48.5 ± 37.6	−19.7 ± 25.8	63.7 ± 7.6%^b^	61.3 ± 56.1%	−1.8 ± 62.5
Fusobacteria	0	0.05 ± 0.1^c^	0.0 ± 0.1^c^	0	0.03 ± 0.03^c^	0.0 ± 0.03^b^	0	0.05 ± 5.5^a^	0.05 ± 5.5	0	13.9 ± 16.1%^a,c,c^	13.9 ± 16.1^b,c^
Proteobacteria	0.63 ± 0.3	1.0 ± 0.7^a,b^	0.6 ± 1.0^b^	0.87 ± 0.25%	5.3 ± 8.7	4.8 ± 9.0	0.52 ± 0.35%	9.1 ± 19.0^a^	8.8 ± 18.7	0.6 ± 0.25%	11.0 ± 38.5%^b^	10.2 ± 38.5^b^
Synergistetes	0.65 ± 0.5	1.0 ± 0.7^b^	0.4 ± 0.8^c^	0.39 ± 0.58%	1.1 ± 0.4^b^	0.55 ± 0.85^b^	0.34 ± 0.35%	0.4 ± 1.0	0.15 ± 1.2	0.71 ± 0.7%	0.1 ± 0.2%^b,b^	−0.7 ± 0.8^b,c^
Verrucomicrobia	0.07 ± 0.0	0.4 ± 0.5^a^	0.4 ± 0.3^b^	0.26 ± 0.53%	2.0 ± 2.3^a,a^	2.0 ± 2.1^a,b^	0.26 ± 0.7%	1.1 ± 3.0	0.8 ± 3.2	0.31 ± 0.6%	0.5 ± 1.0%^a^	0.1 ± 1.2^a^

^a^Statistical difference between groups at the corresponding column and row (*p* < 0.05)

^b^Statistical difference between groups at the corresponding column and row (*p* < 0.01)

^c^Statistical difference between groups at the corresponding column and row (*p* < 0.001)

**Table 3:** Significantly (*p* < 0.05) altered genera in each treatment group compared to the control group

Phylum	Taxon	Change	Group
Actinobacteria	*Adlercreutzia*	↑	Oxaliplatin
Actinobacteria	*Asaccharobacter*	↑	Oxaliplatin
Actinobacteria	*Coriobacteriaceae_NA*	↑	5-FU/oxaliplatin
Actinobacteria	*Olsenella*	↑	Irinotecan
Bacteroidetes	*Bacteroidales_NA_NA*	↓	Irinotecan
Bacteroidetes	*Prevotellaceae_NA*	↓	Oxaliplatin/irinotecan
Bacteroidetes	*Bacteroidia_NA_NA_NA*	↓	Irinotecan
Bacteroidetes	*Bacteroidetes_NA_NA_NA_NA*	↓	Irinotecan
Firmicutes	*Anaerostipes*	↓	Irinotecan
Firmicutes	*Clostridium_XlVa*	↓	Irinotecan
Firmicutes	*Clostridium_XlVb*	↑	Oxaliplatin
Firmicutes	*Dorea*	↑	Oxaliplatin
Firmicutes	*Lachnospiraceae_NA*	↓	Irinotecan
Firmicutes	*Clostridiales_NA_NA*	↓	Irinotecan
Firmicutes	*Clostridium_IV*	↓	Irinotecan
Firmicutes	*Ruminococcaceae_NA*	↓	Irinotecan
Firmicutes	*Clostridia_NA_NA_NA*	↓	Irinotecan
Firmicutes	*Clostridium_XVIII*	↑	Irinotecan
Fusobacteria	*Fusobacterium*	↑	Irinotecan
Proteobacteria	*Parasutterella*	↑	Oxaliplatin
Proteobacteria	*Desulfovibrionaceae_NA*	↑	Irinotecan
Proteobacteria	*Deltaproteobacteria_NA_NA_NA*	↓	Irinotecan
Proteobacteria	*Escherichia/Shigella*	↑	Irinotecan
Synergistetes	*Synergistia_NA_NA_NA*	↓	Irinotecan

*NA* uncertain genera

### Global metabolome variations

**Fig. 4:**
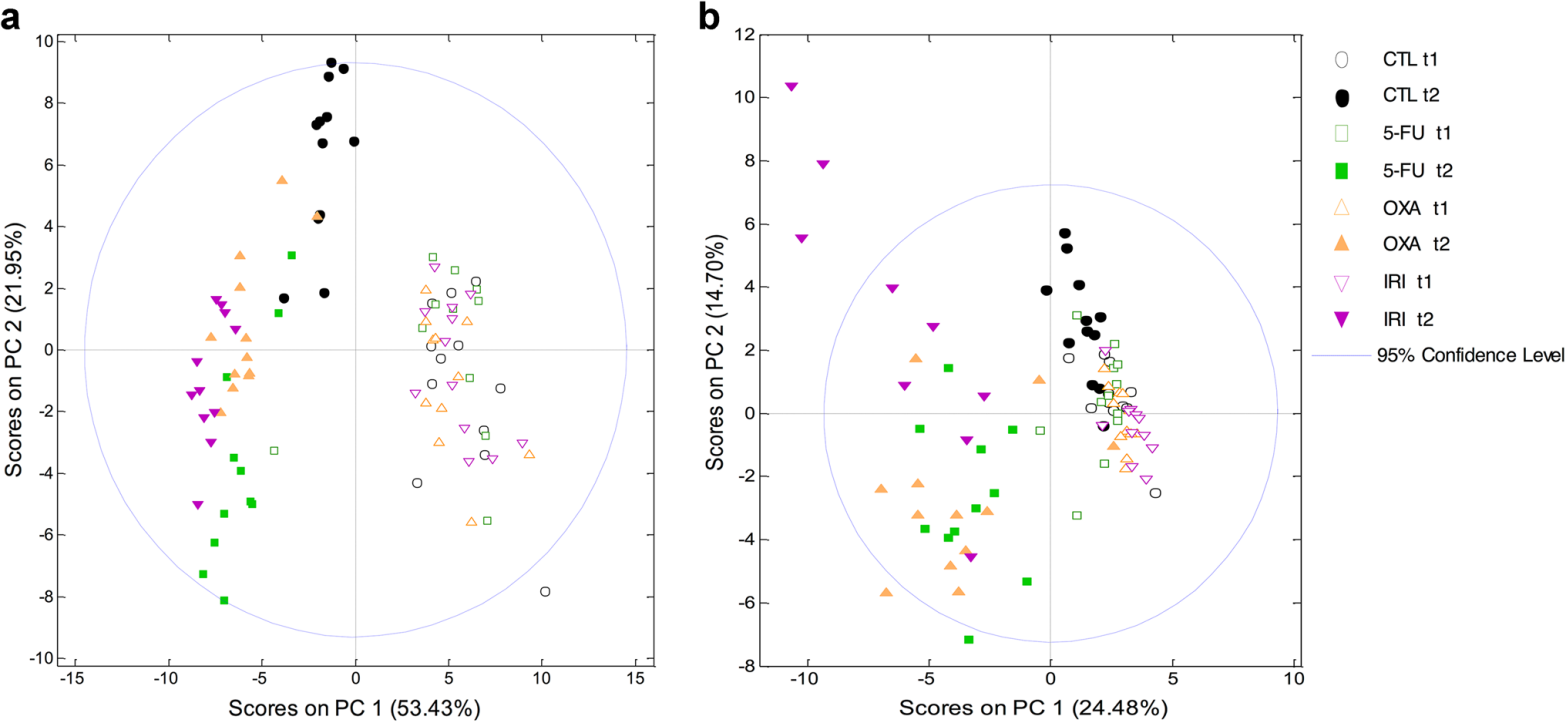
Principal component analysis (PCA) revealed that 5-FU, oxaliplatin, and irinotecan-induced alterations in rat serum metabolome (**a**) and rat urine metabolome (**b**). In both the serum and urine analyses, the baseline samples (*empty shapes*, *t*1) cluster together indicating metabolic similarity between groups. At the end of the experiment (*filled-out shapes*, *t*2), the control serum samples and chemotherapy-treated serum samples cluster separately from the baseline samples suggesting both chemotherapy-induced and time-dependent changes in the metabolome (**a**). In the urine analysis, only the chemotherapy-treated groups cluster separately from baseline indicating only chemotherapy-induced alterations in the metabolome (**b**). *CTL* control, *5-FU* 5-fluorouracil group, *OXA* oxaliplatin group, and *IRI* irinotecan group

**Fig. 5:**
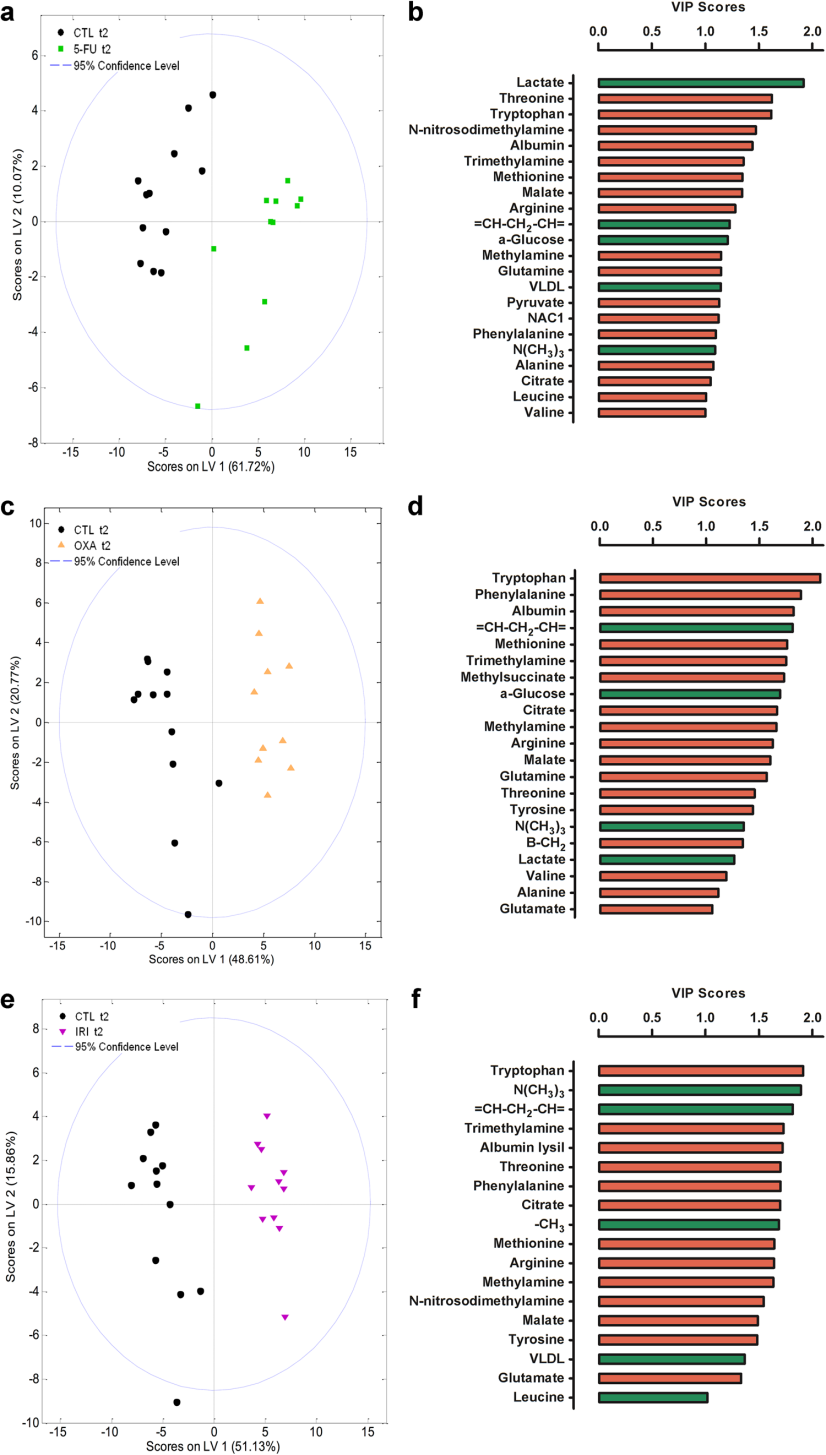
Partial least squares-discriminant analyses (PLS-DA) of serum metabolome showed discrimination between each treatment group and the control group (**a**, **c**, **e**) at the end of the experiment (*t*2). The PLS-DA models were used to calculate variable importance in the projection (VIP) scores for each individual metabolite. VIP scores revealed the individual metabolites that were the most relevant for discrimination (VIP score > 1) between the treatment groups and the control group (**b**, **d**, **f**). The *color* of the *bars* indicates the direction of the metabolic change relative to the control group (*green* significantly increased resonances relative to control group, *red* significantly decreased resonances relative to control group). *CTL* control, *5-FU* 5-fluorouracil group, *OXA* oxaliplatin group, and *IRI* irinotecan group

**Fig. 6:**
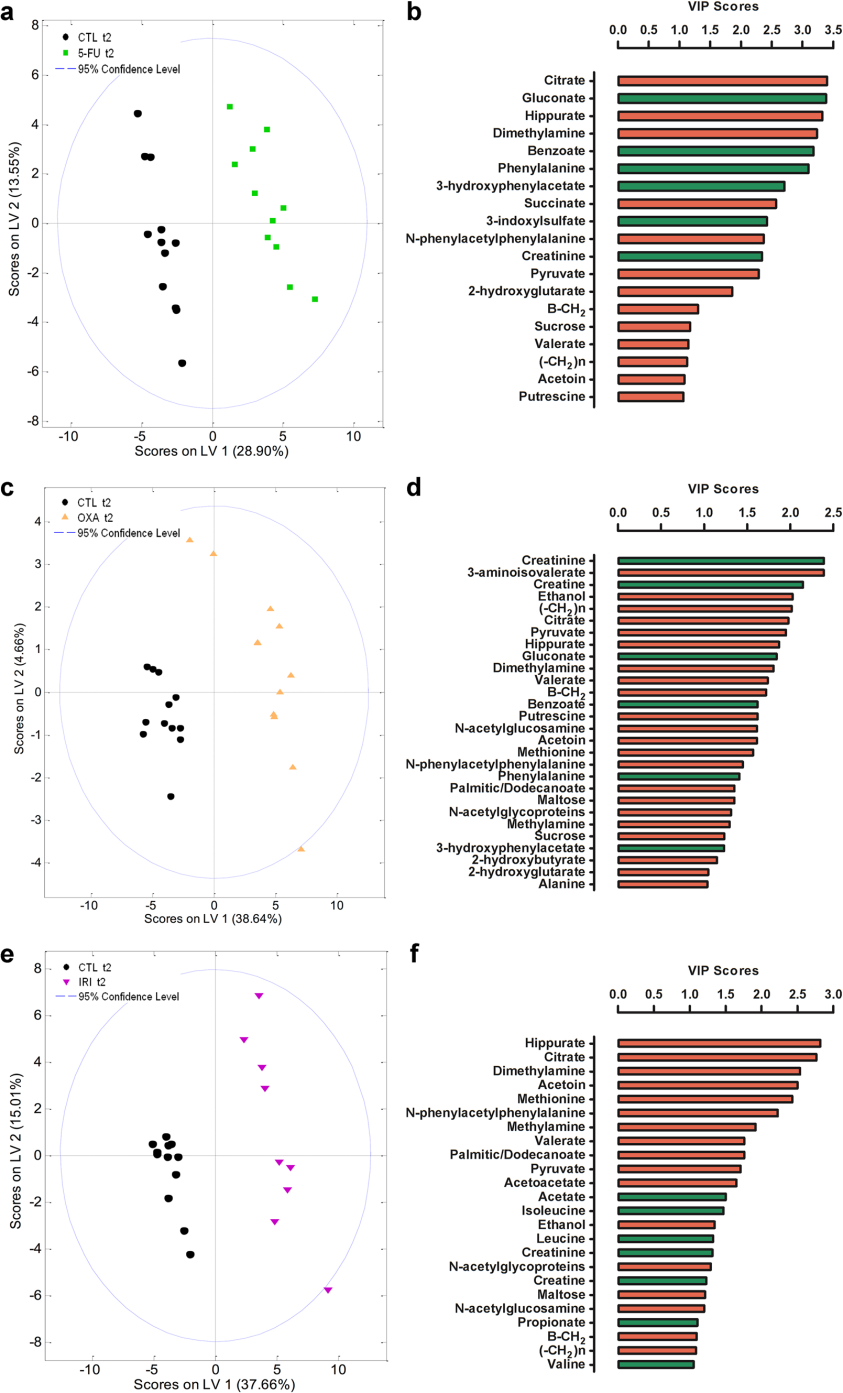
Partial least squares-discriminant analyses (PLS-DA) of urine metabolome showed discrimination between each treatment group and the control group (**a**, **c**, **e**) at the end of the experiment (*t*2). The PLS-DA models were used to calculate variable importance in the projection (VIP) scores for each individual metabolite. VIP scores revealed the individual metabolites that were the most relevant for discrimination (VIP score > 1) between the treatment groups and the control group (**b**, **d**, **f**). The *color* of the *bars* indicates the direction of the metabolic change relative to the control group (*green* significantly increased resonances relative to control group, *red* significantly decreased resonances relative to control group). *CTL* control, *5-FU* 5-fluorouracil group, *OXA* oxaliplatin group, and *IRI* irinotecan group

Metabolomic chemometric analysis by PCA and PLS-DA revealed the chemotherapy-induced metabolic shifts in the rats’ serum and urine. PCA of serum showed three distinct clusters (baselines, control group, and treatment groups) indicating chemotherapeutics-induced as well as time-dependent changes in the metabolome (Fig. 4a). Urine PCA showed only two clusters (baselines and treatment groups), indicating that most of the changes observed in the metabolome were due to the administered chemotherapeutics (Fig. 4b). PLS-DA analyses provided a good discrimination between each treatment group and the control group (Figs. 5, 6). Relevant serum and urine metabolites for discrimination in each treatment group and the direction of the chemotherapeutics-induced change in metabolite levels are visualized in Figs. 5 and 6. All identified metabolites and their resonances are listed in Online Resource 1.

### Correlations between microbiota, metabolites, iohexol permeability, and body weight change

**Fig. 7:**
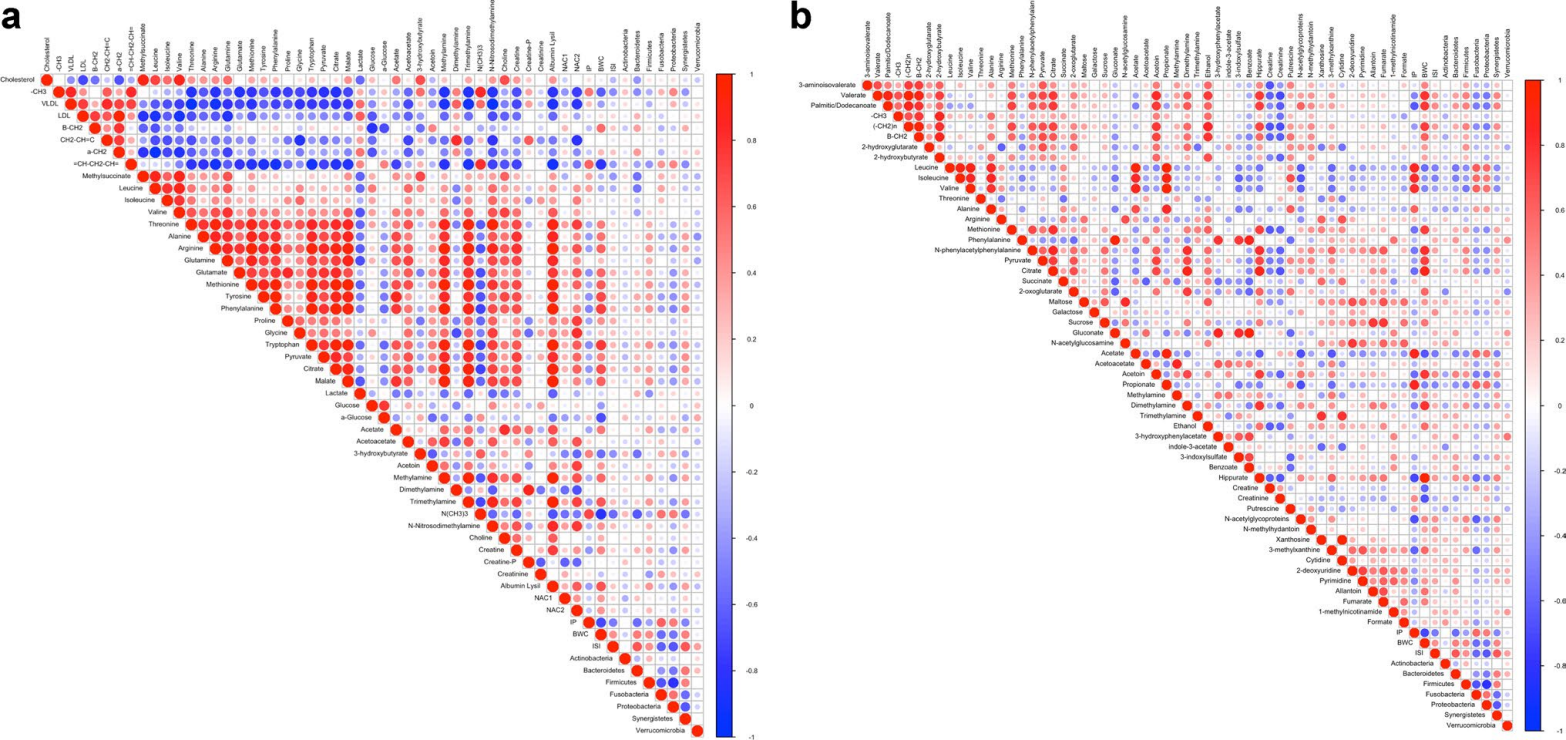
Heat maps showing Pearson’s correlations between intestinal permeability to iohexol (IP), body weight change (BWC), inverse Simpson index (ISI), the relative abundances of bacterial phyla, and metabolite resonances in serum (**a**) and in urine (**b**) at the end of the experiment. The *red circles* indicate a positive correlation and the *blue circles* a negative correlation. Intensity of the *colors* and the size of the *circles* display the strength of the correlation

Pearson’s correlation coefficients were calculated between the relative abundances of each bacterial phylum, inverse Simpson index, intestinal permeability to iohexol, body weight change, serum, and urine metabolites. Correlation matrixes are visualized as heat maps in Fig. 7.

## Discussion

We have previously shown that the 5-FU, oxaliplatin, and irinotecan induce gastrointestinal toxicity that is associated with increased intestinal permeability [15]. The purpose of this study was to characterize how the aforementioned chemotherapeutics affect the composition of fecal microbiota as well as serum and urine metabolome. Our secondary aim was to examine how these changes relate to the gastrointestinal toxicity associated with chemotherapy and to identify possible biomarkers for CIGT.

Overall, our results show that the chemotherapy-associated phenotype is associated with increased relative abundance of bacteria known to produce lipopolysaccharide (LPS), increased levels of serum fatty acids and N(CH_3_)_3_ moieties, and decreased levels of Krebs cycle metabolites and free amino acids. The possible connections and mechanisms behind these changes in the context of CIGT are interesting. For example, our analysis of fecal microbiota revealed that irinotecan treatment induces a significant decrease in microbial diversity. Decreased microbial diversity is a common sign of intestinal dysbiosis that has been linked to multiple GI disorders and inflammatory states [21]. Irinotecan also induced a significant increase in the relative abundance of Proteobacteria, normally a minor component of healthy microbiota, whose increase has been associated with intestinal inflammation in several species [22–25]. The relative abundance of Proteobacteria was also increased in 5-FU and Oxaliplatin groups, but the change from baseline did not reach statistical significance. The previous studies examining the effects of chemotherapeutics on gut microbiota have described similar findings regarding Proteobacteria [26–28]. Lin et al. reported irinotecan-induced increase in the abundance of intestinal Enterobacteriaceae [27], a family of Gram-negative bacteria belonging in the Proteobacteria phylum. The Enterobacteriaceae family includes many known gut pathogens such as *Escherichia/Shigella* spp. whose relative proportion was increased in the Irinotecan group in our study (Table 3). Stringer et al. have also previously described irinotecan-induced increases in intestinal abundance of *Escherichia* spp. [28]. In addition to Proteobacteria, irinotecan also significantly increased the abundance of Fusobacteria. To our knowledge, this is the first study to show that irinotecan-induced gastrointestinal toxicity is associated with increased abundance of fecal Fusobacteria. Fusobacteria are Gram-negative anaerobes that studies have associated with inflammatory bowel disease, development of colorectal cancer, and different infections [29–31]. In addition, in rats, the abundance of fecal Fusobacteria has been shown to correlate with visceral hypersensitivity [32]. Although it is still unclear whether or how Fusobacteria contribute to intestinal inflammation, these findings suggest that Fusobacteria are pathogenic bacteria that promote harmful events in the intestine. Overall, these results raise the question how microbial changes contribute to CIGT. Multiple studies have shown that different chemotherapeutics modulate gut microbiota [26] and that probiotics can ameliorate the severity of CIGT [26, 33, 34]. In addition, antibiotics have shown efficacy in the treatment of irinotecan-induced diarrhea, but whether this effect is due to depletion of gut microbiota [35] or some other mechanism [36] is still up to debate. One of the proposed mechanisms relates to irinotecan’s metabolism by microbial β-glucuronidase [37, 38]. Irinotecan metabolism in the body yields two molecules: irinotecan’s active metabolite 7-ethyl-10-hydroxycamptothecin (SN-38) and glucuronidated metabolite SN-38 (SN-38G). Both metabolites reach the intestinal lumen by biliary excretion. Microbial β-glucuronidase can subsequently convert SN-38G back to its toxic form SN-38 [37] and luminal β-glucuronidase activity has been associated with irinotecan-induced intestinal damage [39, 40]. Previously, Pedroso et al. mono-colonized germ-free mice with either β-glucuronidase-producing *E. coli* or with a mutant *E. coli* strain that does not produce β-glucuronidase [41]. Interestingly, irinotecan caused GI toxicity in both groups, but only mice colonized with β-glucuronidase-producing bacteria exhibited increased intestinal permeability [41]. In our study, irinotecan administration significantly increased the relative abundance of β-glucuronidase-producing bacteria *Escherichia* spp. and induced a significant increase in intestinal permeability. Considering this finding together with Pedroso et al. findings suggests a link between β-glucuronidase-producing bacteria and irinotecan-induced increase in intestinal permeability, but more research is needed to confirm and elucidate the mechanisms behind this connection.

Our results also showed that intestinal permeability to iohexol and body weight loss correlated with microbial diversity and with the abundance of Fusobacteria and Proteobacteria. These findings suggest that microbial dysbiosis plays a role in the pathophysiology of CIGT. Especially, the connection between Proteobacteria and intestinal permeability is interesting. Proteobacteria produce LPS that is known to activate inflammatory processes in the host and the observed increase in intestinal permeability potentially leads to increased LPS leakage into circulation [42]. In addition, the previous studies have shown that chemotherapy can elevate serum LPS levels subsequently activating inflammatory signals [43, 44] and induce cytokine production which may further exacerbate the symptoms of CIGT [45]. We did not directly measure the levels of inflammatory cytokines, but our histological analysis of intestinal tissues revealed significant leucocyte infiltration to lamina propria in all chemotherapy-treated groups suggesting inflammatory activation [15]. The activation of inflammatory processes may also be reflected in the chemotherapeutic metabolome. All treatment groups exhibited significantly elevated values of fatty acid moieties –CH_3_ and =CH–CH_2_–CH=, and VLDL which can be a sign of ongoing inflammation. Inflammatory cytokines and LPS raise the levels of VLDL in circulation by increasing hepatic VLDL production [46]. Based on the previous literature, fatty acid moieties –CH_3_ and =CH–CH_2_–CH= can be assigned to LDL-like lipid particles and polyunsaturated fatty acids (PUFAs), respectively [47, 48]. The increased serum concentrations of these moieties could be the product of inflammation-driven lipolysis and PUFA generation. Martin et al. reported previously that IL-10 −/− mice which spontaneously develop colitis exhibit elevated plasma concentrations of PUFAs. This correlates with inflammatory markers [49] indicating that intestinal inflammation can lead to increased PUFA generation. Interestingly, in patients receiving capecitabine (prodrug of 5-FU), high pre-treatment resonances of –CH_3_ and =CH–CH_2_–CH= moieties seem to predict increased incidences of severe toxicities during the treatment [13]. In our study, the serum levels of these moieties showed a strong correlation with intestinal permeability and body weight loss suggesting that altered serum lipid profile relates to CIGT. This is also supported by the previous studies that have shown that anti-inflammatory and lipid-lowering omega-3-fatty acids can ameliorate CIGT in experimental animals [50] and in colorectal cancer patients [51, 52]. Overall, these findings suggest a link between serum lipids and the pathophysiology of CIGT, but future studies are needed to confirm whether this connection is mediated via inflammatory cytokines.

The chemotherapy-treated groups also showed increased levels of serum N(CH_3_)_3_ moieties and the resonances of these moieties correlated positively with intestinal permeability to iohexol and inversely with body weight change. The N(CH_3_)_3_ moiety is present in several methylamine compounds such as trimethylamine (TMA) and free choline. It also relates to fatty acid metabolism as choline phospholipids contain N(CH_3_)_3_ moieties. Choline phospholipids are major constituents of cell membranes and they are also present in different lipoproteins. In inflammatory states, secretory phospholipase A2 hydrolyses lipoproteins to yield PUFAs and lyso-phospholipidcholines [53] which could explain the concomitant increase of serum levels of =CH–CH_2_–CH= and N(CH_3_)_3_ moieties in our study. Martin et al. observed a similar increase in these two moieties in IL-10 −/− colitis mice, although this was accompanied by a decrease in VLDL serum levels [49]. Backshall et al. also associated elevated pre-treatment serum values of N(CH_3_)_3_ moieties with more severe toxicities during capecitabine treatment and they speculated that this could be due to underlying inflammation [13]. However, the increase in serum values of N(CH_3_)_3_ moieties may also be due to mechanisms relating to the gut microbiota. Gut microbial metabolism converts dietary phosphatidylcholine and choline to TMA [54]. TMA-producing bacteria mainly belong in Actinobacteria, Firmicutes, and Proteobacteria phyla and bacterial species in the Bacteroidetes phylum are poor TMA-producers [54–56]. Interestingly, in our study, the serum levels of N(CH_3_)_3_ moieties showed a strong inverse correlation with the relative abundance of Bacteroidetes and positive correlation with the relative abundances of Proteobacteria. These results suggest that parenterally administered chemotherapeutics can shift the gut microbiota community towards a TMA-producing phenotype. Increase in TMA-producing bacteria can reduce the bioavailability of choline to the host [54] which, in turn, can lead to hepatic steatosis due to the liver’s inability to synthesize phosphatidylcholine necessary for the assembly and secretion of VLDLs [57]. However, we observed a significant increase in the serum levels of VLDL indicating sufficient choline availability and liver function. Nonetheless, the connections between elevated serum resonances of N(CH_3_)_3_ moieties, gut microbiota, and CIGT are interesting.

We also observed significant changes in energy metabolism in the treatment groups. All treatment groups exhibited significantly decreased serum and urinary levels of Krebs cycle metabolites. However, how these changes relate to chemotherapy-induced toxicities is unclear. First, Connor et al. have shown that Krebs cycle metabolites are prone to changes in response to body weight changes and thus represent non-specific metabolic responses to toxicities [58]. Second, urinary concentrations of Krebs cycle metabolites decrease during fasting [59, 60]. It is important to note that the animals were under stressful conditions (metabolic caging and chemotherapy) during the study which can affect food consumption and shift energy metabolism towards catabolic pathways. This is an obvious limitation in this study but as the baseline samples and the samples from the control animals were also collected under or after metabolic caging, some of the observed changes in the metabolome can be attributed to the chemotherapeutics. For example, chemotherapy-induced damage to the intestine may impair nutrient absorption and thus lead to reduced nutrient intake and availability. Under nutrient depletion, the body maintains its energetic homeostasis by increased fatty acid utilization and ketogenesis, increased glycogenolysis and gluconeogenesis, and by muscle protein breakdown. These processes induce several metabolic alterations and some of them were also evident in our study. Regarding ketogenesis, the Irinotecan group exhibited a small but significant increase in the serum values of 3-hydroxybutyrate. The serum levels of another ketone body acetoacetate did not differ between the groups, suggesting that ketogenesis was not overly active in the treatment groups despite their increased levels of certain serum fatty acid moieties. Regarding glucose metabolism, 5-FU and oxaliplatin groups exhibited significantly elevated serum levels of lactate indicating a shift from oxidative phosphorylation to anaerobic energy production. Anaerobic energy production in hypoxic conditions downregulates Krebs cycle activity via mechanisms that also involve ROS sensing and generation [61]. Chemotherapy-induced ROS generation may thus contribute to the observed alterations in Krebs cycle metabolites. We also observed changes in skeletal muscle energy metabolism as all treatment groups exhibited significantly increased values of urinary creatinine. Creatinine is the degradation product of creatine phosphate and increased excretion of creatinine could thus signal increased creatine phosphate utilization and possible energy depletion in the skeletal muscles [59]. Overall, the observed alterations in energetic metabolites suggest that the energetic homeostasis is maintained via catabolic pathways. However, these changes are most likely not specific to the studied chemotherapeutics but represent non-specific metabolic responses to the treatments.

Energy metabolism also relates to protein and amino acid metabolism. Muscle protein breakdown leads to the release of amino acids that the body can subsequently convert to energetic metabolites. In the irinotecan group, we observed significantly elevated urinary levels of branch-chained amino acids leucine, isoleucine, and valine indicating increased muscle protein catabolism [59]. Overall, however, the treatment groups exhibited decreased serum values of multiple amino acids including tryptophan, threonine, phenylalanine, arginine, methionine, alanine, tyrosine, glutamine, and glutamate. Several mechanisms can explain this finding. First, tryptophan, threonine, phenylalanine, and methionine are essential amino acids that must be obtained from diet, so their decrease could be a sign of reduced nutrient availability. Second, under energy deprivation, the body can shuttle these amino acids to gluconeogenesis to sustain adequate blood glucose levels. Third, some of these amino acids are also involved in inflammatory processes. For example, pro-inflammatory cytokines induce indoleamine 2,3-dioxygenase (IDO)-mediated tryptophan catabolism and studies have linked low serum tryptophan concentrations to multiple inflammatory diseases [62–64]. Intestinal inflammation can also reduce serum levels of glutamate and glutamine [65, 66], although contradictory findings also exist [49]. Nonetheless, glutamine is an important energy substrate for enterocytes and the observed decrease in glutamine levels in our study could be the result of increased glutamine metabolism in the enterocytes. Glutamine supplementation can alleviate intestinal inflammation in experimental animals [66] and it has also had some success in reducing the symptoms of CIGT, but its clinical relevance is still unclear [67]. Inflammatory cytokines also play a role in arginine metabolism. Myeloid cells use arginine to generate nitric oxide (NO) in the presence of inflammatory stimuli and studies have shown that both arginine and NO are important mediators of normal gastrointestinal function [68]. Studies have also shown that NO is an important inflammatory mediator of 5-FU [69] and irinotecan [70] induced mucositis. Thus, the decreased levels of arginine in our study could result from increased NO production due to chemotherapy-induced inflammation.

In addition to modulating gut microbiota, the studied chemotherapeutics also caused significant alterations in the levels of microbiota-associated metabolite hippurate. Hippurate, the glycine-conjugate of benzoic acid, is a major mammalian–microbial cometabolite whose excretion depends on gut microbiota in that germ-free animals do not excrete hippurate [71]. Gut microbiota metabolizes dietary aromatic compounds to benzoic acid that the liver and kidney subsequently conjugate with glycine to form hippurate [72]. We observed a significant decrease in urinary hippurate levels in all treatment groups and this decrease correlated strongly with intestinal permeability to iohexol and body weight loss. Although hippurate excretion is susceptible to body weight changes and could thus represent a non-specific response to drug toxicities [58], we observed an interesting trend in hippurate formation. The control group exhibited low levels of urinary benzoate together with high levels of hippurate indicating a normal pattern of hippurate formation and excretion [71]. The 5-FU and oxaliplatin groups, however, showed elevated levels of urinary benzoate and decreased levels of hippurate suggesting microbial production of benzoate but compromised hippurate formation. Abnormal hippurate formation also appeared in the Irinotecan group, where the animals excreted benzoate at a similar level as the control group, but this was accompanied by very low urinary levels of hippurate. This pattern hints that irinotecan affects microbial benzoate metabolism and subsequent hippurate formation. This could be related to the irinotecan-induced intestinal dysbiosis as dysbiosis of the gut microbiota has been associated with decreased urinary concentrations of hippurate in patients with IBDs [72]. In our study, urinary hippurate levels correlated positively with fecal microbial diversity suggesting that decreased hippurate excretion may be a good indicator of disturbed intestinal microbiota homeostasis in CIGT.

In conclusion, commonly used chemotherapeutics 5-FU, oxaliplatin, and irinotecan induce several microbial and metabolic changes which may play a role in the pathophysiology of CIGT. Alterations in the composition of fecal microbiota and decreased levels of urinary hippurate indicate intestinal dysbiosis that together with increased intestinal permeability activates of inflammatory processes. This activation is reflected in the metabolome via increased serum levels of PUFAs and N(CH_3_)_3_ moieties and decreased serum levels of tryptophan, glutamine, and arginine. However, the causality of these changes in the context of CIGT is still unclear and warrants more research. In addition, several metabolic alterations could be the results of a non-specific response to the studied chemotherapeutic and more detailed analyses are needed to separate the effects of stress and nutrition on the chemotherapy-induced changes of the metabolome.

## Electronic supplementary material

Below is the link to the electronic supplementary material.

Supplementary material 1 (DOCX 96 kb)
