# Supplementary material for: Chemotherapy-induced gastrointestinal toxicity is associated with changes in serum and urine metabolome and fecal microbiota in male Sprague–Dawley rats
Source: Cancer Chemother Pharmacol. 2017 Jun 23;80(2):317–32. doi: 10.1007/s00280-017-3364-z (PMC5532424; doi:10.1007/s00280-017-3364-z)
Supplement: Supplementary file 1 — Supplementary material 1 (DOCX 96 kb) [file 280_2017_3364_MOESM1_ESM.docx]

**Supplementary material 1**

**CHEMOTHERAPY-INDUCED GASTROINTESTINAL TOXICITY IS ASSOCIATED WITH CHANGES IN SERUM AND URINE METABOLOME AND FECAL MICROBIOTA IN MALE SPRAGUE-DAWLEY RATS**

*Richard A. Forsgård*^1^*, Vannina G Marrachelli*^2^, *Katri Korpela*^3^, *Rafael Frias*^4,5^, *Maria Carmen Collado*^6^ *Riitta Korpela*^1^*, Daniel Monleon*^2^, *Thomas Spillmann*^7^*, Pia Österlund*^8,9^

^1^Pharmacology, Faculty of Medicine, University of Helsinki, Helsinki, Finland

^2^Health research Institute INCLIVA, Metabolomics and Molecular Imaging Lab, Valencia, Spain

^3^Immunobiology Research Program, Department of Bacteriology and Immunology, University of Helsinki, Helsinki, Finland

^4^Central Animal Laboratory, University of Turku, Turku, Finland

^5^Comparative Medicine, Karolinska Institutet, Stockholm, Sweden

^6^Institute of Agrochemistry and Food Technology, National Research Council (IATA-CSIC), Valencia, Spain

^7^Department of Equine and Small Animal Medicine, Faculty of Veterinary Medicine, University of Helsinki, Helsinki, Finland

^8^Department of Oncology, University of Helsinki and Helsinki University Hospital, Helsinki, Finland

^9^Department of Oncology, Tampere University Hospital, Tampere, Finland

**Corresponding author:** Richard Forsgård, Faculty of Medicine, Pharmacology, P.O. Box 63, FI-00014 University of Helsinki, Finland, richard.forsgard@helsinki.fi Telephone: +358-50-448-2227 Fax: +358-9-4546168

**Table S1** The relative abundances (% of all reads) of all identified genus level taxa in the rats’ feces at baseline (t1) and at the end of experiment (t2). All data are listed as mean ± standard deviation.

|  |  | **Control** | | | **5-Fluorouracil** | | | **Oxaliplatin** | | | **Irinotecan** | | |
| --- | --- | --- | --- | --- | --- | --- | --- | --- | --- | --- | --- | --- | --- |
| **Phyla** | **Taxon** | **t1** | **t2** | **Δ** | **t1** | **t2** | **Δ** | **t1** | **t2** | **Δ** | **t1** | **t2** | **Δ** |
| **Actinobacteria** | *Arthrobacter* | 0.02 ± 0.02 | 0.09 ± 0.15 | 0.07 ± 0.15 | 0.01 ± 0.02 | 0.10 ± 0.19 | 0.09 ± 0.19 | 0.02 ± 0.02 | 0.27 ± 0.54 | 0.25 ± 0.55 | 0.02 ± 0.03 | 0.07 ± 0.20 | 0.05 ± 0.21 |
| **Actinobacteria** | *Kocuria* | 0.02 ± 0.02 | 0.06 ± 0.06^b^ | 0.03 ± 0.05 | 0.01 ± 0.02^a^ | 0.0 ± 0.02^a,b,b^ | -0.01 ± 0.02 | 0.04 ± 0.03^a^ | 0.08 ± 0.08^b^ | 0.03 ± 0.09 | 0.01 ± 0.05 | 0.04 ± 0.06^a^ | 0.04 ± 0.1 |
| **Actinobacteria** | *Actinomycetales NA_NA* | 0.03 ± 0.02 | 0.05 ± 0.03 | 0.02 ± 0.04 | 0.03 ± 0.02 | 0.06 ± 0.04 | 0.03 ± 0.04 | 0.03 ± 0.02 | 0.03 ± 0.04 | -0.0 ± 0.05 | 0.04 ± 0.02 | 0.07 ± 0.09 | 0.04 ± 0.10 |
| **Actinobacteria** | *Bifidobacterium* | 7.2 ± 7.0 | 0.21 ± 0.34 | -6.7 ± 6.4 | 1.5 ± 1.4^a,b^ | 0.1 ± 3.6 | -0.71 ± 2.1 | 10.5 ± 10.0^a^ | 0.28 ± 0.77 | -9.9 ± 9.8 | 11.0 ± 7.5^b^ | 0.79 ± 6.5 | -7.3 ± 9.5 |
| **Actinobacteria** | *Adlercreutzia* | 0.01 ± 0.01 | 0.01 ± 0.02^a,b^ | -0.01 ± 0.01^b^ | 0.01 ± 0.0 | 0.04 ± 0.02^a^ | 0.03 ± 0.02 | 0.01 ± 0.0 | 0.05 ± 0.05^b^ | 0.04 ± 0.05^b^ | 0.01 ± 0.02 | 0.02 ± 0.04 | 0.01 ± 0.03 |
| **Actinobacteria** | *Asaccharobacter* | 0.05 ± 0.03 | 0.06 ± 0.05^a^ | 0.02 ± 0.07^b^ | 0.05 ± 0.03 | 0.13 ± 0.07 | 0.06 ± 0.06 | 0.04 ± 0.03 | 0.2 ± 0.23^a^ | 0.15 ± 0.21^a,b^ | 0.05 ± 0.03 | 0.09 ± 0.1 | 0.05 ± 0.06^a^ |
| **Actinobacteria** | *Collinsella* | 0.0 ± 0.0 | 0.01 ± 0.01 | 0.0 ± 0.01 | 0.01 ± 0.01 | 0.0 ± 0.01 | -0.0 ± 0.01 | 0 | 0.02 ± 0.04 | 0.02 ± 0.04 | 0 | 0.0 ± 0.01 | 0.0 ± 0.01 |
| **Actinobacteria** | *Eggerthella* | 0.0 ± 0.0 | 0.0 ± 0.0 | 0.0 ± 0.0 | 0.0 ± 0.0 | 0.0 ± 0.0 | 0.0 ± 0.0 | 0 | 0.01 ± 0.02 | 0.01 ± 0.02 | 0 | 0 | 0 |
| **Actinobacteria** | *Coriobacteriaceae NA* | 0.05 ± 0.01 | 0.06 ± 0.03^a,b^ | 0.01 ± 0.05^a,a^ | 0.03 ± 0.02 | 0.16 ± 0.06^b,b^ | 0.12 ± 0.04^a,b^ | 0.03 ± 0.02 | 0.14 ± 0.17^a,a^ | 0.11 ± 0.16^a,b^ | 0.04 ± 0.04 | 0.05 ± 0.08^a,b^ | 0.02 ± 0.05^b,b^ |
| **Actinobacteria** | *Olsenella* | 0.03 ± 0.03 | 0.02 ± 0.02^b^ | -0.02 ± 0.05^a^ | 0.02 ± 0.03^a,b^ | 0.03 ± 0.12^a^ | 0.01 ± 0.06 | 0.09 ± 0.14^a^ | 0.02 ± 0.07^a^ | -0.05 ± 0.09^b^ | 0.1 ± 0.29^b^ | 0.23 ± 0.74^a,a,b^ | 0.15 ± 0.65^a,b^ |
| **Actinobacteria** | *Coriobacteriales NA_NA* | 0.15 ± 0.06 | 0.22 ± 0.14 | 0.07 ± 0.13 | 0.19 ± 0.16 | 0.36 ± 0.25 | 0.16 ± 0.34 | 0.20 ± 0.09 | 0.17 ± 0.19 | -0.02 ± 0.20 | 0.18 ± 0.07 | 0.20 ± 0.21 | 0.01 ± 0.23 |
| **Actinobacteria** | *Actinobacteria NA_NA_NA* | 0.09 ± 0.09 | 0.07 ± 0.06 | -0.02 ± 0.07 | 0.10 ± 0.08 | 0.17 ± 0.13 | 0.08 ± 0.11 | 0.11 ± 0.07 | 0.10 ± 0.11 | -0.0 ± 0.13 | 0.14 ± 0.12 | 0.18 ± 0.21 | 0.04 ± 0.24 |
| **Bacteroidetes** | *Bacteroides* | 1.0 ± 0.57 | 1.8 ± 1.1 | 0.79 ± 1.2 | 1.0 ± 1.0 | 3.8 ± 1.8 | 2.7 ± 2.1 | 0.78 ± 0.91 | 4.5 ± 2.6 | 3.7 ± 3.0 | 0.57 ± 0.43 | 4.3 ± 4.8 | 3.7 ± 5.0 |
| **Bacteroidetes** | *Bacteroidales NA_NA* | 0.5 ± 0.45 | 1.0 ± 0.37^b^ | 0.55 ± 0.71^b^ | 0.6 ± 0.42 | 1.2 ± 0.91^b^ | 0.43 ± 1.2^b^ | 0.62 ± 0.68 | 1.1 ± 0.91^b^ | 0.48 ± 0.68^b^ | 0.77 ± 0.48 | 0.22 ± 0.44^b,b,b^ | -0.44 ± 0.29^b,b,b^ |
| **Bacteroidetes** | *Butyricimonas* | 0.05 ± 0.02 | 0.16 ± 0.14 | 0.11 ± 0.15 | 0.05 ± 0.03 | 0.27 ± 0.11 | 0.22 ± 0.10 | 0.07 ± 0.06 | 0.25 ± 0.15 | 0.18 ± 0.18 | 0.08 ± 0.05 | 0.23 ± 0.29 | 0.15 ± 0.31 |
| **Bacteroidetes** | *Porphyromonadaceae NA* | 0.01 ± 0.01 | 0.04 ± 0.03 | 0.03 ± 0.04 | 0.02 ± 0.01 | 0.05 ± 0.03 | 0.04 ± 0.03 | 0.01 ± 0.01 | 0.05 ± 0.04 | 0.04 ± 0.05 | 0.02 ± 0.01 | 0.05 ± 0.06 | 0.03 ± 0.06 |
| **Bacteroidetes** | *Parabacteroides* | 0.29 ± 0.25 | 0.48 ± 0.14 | 0.19 ± 0.33 | 0.19 ± 0.13 | 1.1 ± 0.49 | 0.88 ± 0.53 | 0.30 ± 0.26 | 1.4 ± 1.1 | 1.1 ± 1.1 | 0.19 ± 0.11 | 2.0 ± 2.6 | 1.8 ± 2.6 |
| **Bacteroidetes** | *Prevotellaceae NA* | 1.7 ± 1.7 | 3.1 ± 4.3^c^ | 0.18 ± 5.7^a,c^ | 1.6 ± 2.5 | 2.0 ± 1.3^b^ | -0.01 ± 2.5 | 1.4 ± 1.4 | 0.86 ± 1.1 | -0.7 ± 1.0^a^ | 1.1 ± 1.8 | 0.02 ± 0.07^b,c^ | -1.0 ± 1.7^c^ |
| **Bacteroidetes** | *Alistipes* | 0.28 ± 0.38 | 0.66 ± 0.32^a^ | 0.39 ± 0.5 | 0.34 ± 0.32 | 0.47 ± 0.35 | 0.05 ± 0.4 | 0.27 ± 0.16 | 0.83 ± 0.73^b^ | 0.51 ± 0.66^a^ | 0.41 ± 0.43 | 0.18 ± 0.28^a,b^ | -0.9 ± 0.56^a^ |
| **Bacteroidetes** | *Bacteroidia NA_NA_NA* | 13.4 ± 15.3 | 13.6 ± 7.8^b^ | 3.8 ± 13.7^c^ | 8.8 ± 5.4 | 17.2 ± 4.7^c^ | 7.3 ± 5.4^c^ | 10.9 ± 7.1 | 12.7 ± 5.6^a^ | 2.0 ± 11.5^c^ | 13.0 ± 7.6 | 0.71 ± 1.9^a,b,c^ | -12.8 ± 9.1^c,c,c^ |
| **Bacteroidetes** | *Flavobacteria NA_NA_NA* | 0.03 ± 0.02 | 0.02 ± 0.01^b^ | -0.01 ± 0.03 | 0.02 ± 0.02^b^ | 0.08 ± 0.04^a,b^ | 0.06 ± 0.07 | 0.02 ± 0.02^a^ | 0.03 ± 0.04^a^ | 0.0 ± 0.05 | 0.04 ± 0.03^a,b^ | 0.01 ± 0.11 | -0.2 ± 0.1 |
| **Bacteroidetes** | *Bacteroidetes NA_NA_NA_NA* | 7.0 ± 4.1 | 7.5 ± 3.9^b^ | 0.73 ± 5.8^b^ | 3.5 ± 2.8 | 10.5 ± 4.5^c^ | 5.4 ± 6.8^c^ | 4.5 ± 3.2 | 7.4 ± 7.3^a^ | 1.3 ± 6.1^b^ | 6.2 ± 1.9 | 1.3 ± 3.0^a,b,c^ | -4.6 ± 2.2^b,b,c^ |
| **Firmicutes** | *Enterococcus* | 0.04 ± 0.31 | 0.0 ± 0.01^c^ | -0.2 ± 0.3 | 0.08 ± 43.3 | 0.02 ± 0.13^b^ | -0.07 ± 43.1 | 0.02 ± 0.14 | 0.02 ± 0.05^b^ | 0.0 ± 0.1 | 0.01 ± 0.03 | 0.54 ± 1.18^a,b,b^ | 0.52 ± 1.2 |
| **Firmicutes** | *Lactobacillus* | 19.5 ± 10.3 | 14.3 ± 7.4 | -5.3 ± 11.9 | 17.9 ± 10.5 | 17.9 ± 7.8 | -0.06 ± 9.9 | 20.8 ± 8.1 | 7.9 ± 7.0 | -12.9 ± 8.7 | 17.4 ± 5.3 | 15.5 ± 15.2 | -1.9 ± 14.3 |
| **Firmicutes** | *Lactococcus* | 0.0 ± 0.01 | 0.12 ± 0.33 | 0.11 ± 0.32 | 0 | 0.04 ± 0.05 | 0.04 ± 0.05 | 0.0 ± 0.01 | 0.04 ± 0.06 | 0.03 ± 0.06 | 0 | 0.09 ± 0.16 | 0.09 ± 0.16 |
| **Firmicutes** | *Streptococcus* | 0.0 ± 0.0 | 0.02 ± 0.03 | 0.02 ± 0.03 | 0.01 ± 0.01 | 0.0 ± 0.0 | -0.0 ± 0.01 | 0.0 ± 0.0 | 0.01 ± 0.03 | 0.01 ± 0.03 | 0.0 ± 0.01 | 0.01 ± 0.01 | 0.0 ± 0.01 |
| **Firmicutes** | *Clostridium sensu stricto* | 4.2 ± 2.8^b^ | 0.22 ± 0.6 | -4.0 ± 2.9 | 3.5 ± 5.1^b^ | 0.17 ± 0.3 | -3.3 ± 5.1 | 4.6 ± 4.4 | 0.43 ± 0.7 | -4.1 ± 4.3 | 9.7 ± 5.6^b,b^ | 1.4 ± 9.4 | -6.9 ± 10.4 |
| **Firmicutes** | *Eubacterium* | 0.49 ± 0.43 | 0.62 ± 0.41^a^ | 0.06 ± 0.32 | 0.35 ± 0.45 | 0.34 ± 0.4 | -0.03 ± 0.61 | 0.46 ± 0.38 | 0.56 ± 0.94^a^ | 0.27 ± 0.74^a^ | 0.36 ± 0.24 | 0.17 ± 0.23^a,a^ | -0.27 ± 0.29^a^ |

a = statistical difference between groups at the corresponding column and row (p < 0.05)

b = statistical difference between groups at the corresponding column and row (p < 0.01)

c = statistical difference between groups at the corresponding column and row (p < 0.001)

NA = uncertain genera

**Table S1** continues…

|  |  | **Control** | | | **5-Fluorouracil** | | | **Oxaliplatin** | | | **Irinotecan** | | |
| --- | --- | --- | --- | --- | --- | --- | --- | --- | --- | --- | --- | --- | --- |
| **Phyla** | **Taxon** | **t1** | **t2** | **Δ** | **t1** | **t2** | **Δ** | **t1** | **t2** | **Δ** | **t1** | **t2** | **Δ** |
| **Firmicutes** | *Eubacteriaceae NA* | 0.09 ± 0.24 | 0.15 ± 0.31 | 0.05 ± 0.42 | 0.11 ± 0.16 | 0.10 ± 0.16 | -0.01 ± 0.26 | 0.10 ± 0.16 | 0.11 ± 0.15 | 0.01 ± 0.23 | 0.03 ± 0.04 | 0.12 ± 0.17 | 0.09 ± 0.19 |
| **Firmicutes** | *Anaerostipes* | 0.02 ± 0.04 | 0.06 ± 0.15^c^ | 0.04 ± 0.11^a^ | 0.05 ± 0.07^a^ | 0.06 ± 0.14^c^ | -0.2 ± 0.09 | 0.02 ± 0.02 | 0.05 ± 0.05^a^ | 0.03 ± 0.05 | 0.01 ± 0.02^a^ | 0^a,c,c^ | -0.01 ± 0.02^a^ |
| **Firmicutes** | *Clostridium XIVa* | 1.2 ± 0.74^b^ | 1.1 ± 0.57^c^ | 0.05 ± 1.6^b^ | 0.8 ± 1.4 | 0.57 ± 0.57 | 0.05 ± 1.7 | 0.46 ± 0.48 | 0.9 ± 0.78^b^ | 0.28 ± 0.65^b^ | 0.21 ± 0.42^b^ | 0.15 ± 0.20^b,c^ | -0.07 ± 0.59^b,b^ |
| **Firmicutes** | *Clostridium XIVb* | 0.01 ± 0.02 | 0.02 ± 0.02^a^ | 0.0 ± 0.02^a^ | 0.02 ± 0.02 | 0.01 ± 0.03^b^ | -0.01 ± 0.04 | 0.01 ± 0.01 | 0.18 ± 1.1^a,b,c^ | 0.17 ± 1.1^a,b^ | 0.0 ± 0.02 | 0.0 ± 0.0^c^ | 0.0 ± 0.01^b^ |
| **Firmicutes** | *Coprococcus* | 0.36 ± 0.82 | 0.15 ± 0.15 | -0.21 ± 0.84 | 0.04 ± 0.04 | 0.12 ± 0.14 | 0.09 ± 0.14 | 0.02 ± 0.03 | 0.05 ± 0.07 | 0.03 ± 0.08 | 0.01 ± 0.02 | 0.10 ± 0.14 | 0.09 ± 0.14 |
| **Firmicutes** | *Dorea* | 0.0 ± 0.01 | 0.0 ± 0.01^a^ | 0.0 ± 0.0^a^ | 0.0 ± 0.01 | 0.0 ± 0.01^b^ | 0.0 ± 0.01 | 0.0 ± 0.0 | 0.06 ± 0.38^a,b^ | 0.06 ± 0.39^a,a^ | 0.0 ± 0.0 | 0.01 ± 0.07 | 0.0 ± 0.06^a^ |
| **Firmicutes** | *Hespellia* | 0.25 ± 0.25 | 0.32 ± 0.19 | 0.07 ± 0.35 | 0.45 ± 0.28 | 0.15 ± 0.06 | -0.30 ± 0.30 | 0.28 ± 0.17 | 0.29 ± 0.21 | 0.01 ± 0.32 | 0.31 ± 0.13 | 0.14 ± 0.14 | -0.17 ± 0.16 |
| **Firmicutes** | *Lachnospiracea incertae sedis* | 2.0 ± 1.5 | 1.3 ± 0.61 | -0.72 ± 1.7 | 1.1 ± 0.86 | 0.91 ± 0.36 | -0.22 ± 0.82 | 0.81 ± 0.42 | 1.5 ± 0.41 | 0.73 ± 0.67 | 0.71 ± 0.45 | 1.3 ± 1.6 | 0.63 ± 1.5 |
| **Firmicutes** | *Moryella* | 0.18 ± 2.4^a^ | 0.35 ± 0.89^b^ | 0.24 ± 0.89 | 0.17 ± 0.3 | 0.3 ± 0.66^a^ | 0.02 ± 0.76 | 0.05 ± 0.11 | 0.48 ± 0.55^b^ | 0.39 ± 0.56 | 0.03 ± 0.34^a^ | 0.01 ± 0.11^a,b,b^ | -0.03 ± 0.30 |
| **Firmicutes** | *Lachnospiraceae NA* | 1.7 ± 2.1 | 2.4 ± 2.8^c^ | 1.4 ± 4.5^b^ | 1.4 ± 1.7 | 0.98 ± 0.78^a^ | -0.09 ± 2.0 | 1.2 ± 1.5 | 1.3 ± 2.1^b^ | -0.33 ± 2.5 | 0.37 ± 0.66 | 0.03 ± 0.06^a,b,c^ | -0.36 ± 0.63^b^ |
| **Firmicutes** | *Pseudobutyrivibrio* | 0.05 ± 0.05 | 0.03 ± 0.02 | -0.02 ± 0.06 | 0.04 ± 0.03 | 0.05 ± 0.03 | 0.01 ± 0.02 | 0.03 ± 0.01 | 0.03 ± 0.02 | -0.0 ± 0.02 | 0.03 ± 0.03 | 0.03 ± 0.03 | -0.0 ± 0.06 |
| **Firmicutes** | *Robinsoniella* | 0.0 ± 0.01 | 0.0 ± 0.0 | -0.0 ± 0.01 | 0.0 ± 0.0 | 0 | -0.0 ± 0.0 | 0.0 ± 0.0 | 0.0 ± 0.01 | 0.0 ± 0.01 | 0.0 ± 0.0 | 0.01 ± 0.03 | 0.01 ± 0.03 |
| **Firmicutes** | *Roseburia* | 0.0 ± 0.0 | 0.0 ± 0.01 | 0.0 ± 0.01 | 0.0 ± 0.01 | 0.0 ± 0.01 | -0.0 ± 0.02 | 0 | 0.01 ± 0.02 | 0.01 ± 0.02 | 0.0 ± 0.0 | 0.0 ± 0.01 | 0.0 ± 0.01 |
| **Firmicutes** | *Clostridiales NA_NA* | 3.4 ± 3.7 | 8.2 ± 7.8^c^ | 4.4 ± 8.6^b^ | 4.4 ± 4.2 | 4.2 ± 4.6 | 0.88 ± 8.2 | 3.9 ± 3.0 | 5.4 ± 9.9^a^ | 2.3 ± 5.8^a^ | 2.1 ± 1.7 | 1.5 ± 2.2^a,c^ | -1.4 ± 3.1^a,b^ |
| **Firmicutes** | *Clostridium XI* | 7.6 ± 3.4 | 4.3 ± 4.2 | -3.3 ± 5.9 | 6.9 ± 5.8 | 2.7 ± 2.0 | -4.2 ± 6.0^a^ | 10.7 ± 3.6 | 2.3 ± 2.8 | -8.4 ± 5.3^a^ | 11.2 ± 2.4 | 11.7 ± 11.9 | 0.46 ± 12.5^a,a^ |
| **Firmicutes** | *Peptostreptococcaceae NA* | 0.02 ± 0.02 | 0.05 ± 0.08 | 0.03 ± 0.06 | 0.03 ± 0.02 | 0.05 ± 0.06 | 0.03 ± 0.06 | 0.02 ± 0.02 | 0.08 ± 0.04 | 0.05 ± 0.03 | 0.02 ± 0.03 | 0.03 ± 0.03 | 0.01 ± 0.05 |
| **Firmicutes** | *Clostridium IV* | 0.20 ± 0.12 | 0.24 ± 0.16^b^ | 0.08 ± 0.15^a^ | 0.18 ± 0.25 | 0.21 ± 0.15^a^ | 0.02 ± 0.23 | 0.10 ± 0.14 | 0.33 ± 0.31^c^ | 0.19 ± 0.28^c^ | 0.12 ± 0.26 | 0.07 ± 0.08^a,b,c^ | -0.07 ± 0.27^a,c^ |
| **Firmicutes** | *Faecalibacterium* | 0.01 ± 0.01 | 0.02 ± 0.05 | 0.02 ± 0.04 | 0.01 ± 0.02 | 0.01 ± 0.02 | 0.0 ± 0.03 | 0.01 ± 0.0 | 0.04 ± 0.10 | 0.04 ± 0.10 | 0.01 ± 0.01 | 0.01 ± 0.02 | -0.0 ± 0.02 |
| **Firmicutes** | *Flavonifractor* | 0 | 0 | 0 | 0 | 0.0 ± 0.0 | 0.0 ± 0.0 | 0 | 0.01 ± 0.02 | 0.01 ± 0.02 | 0 | 0 | 0 |
| **Firmicutes** | *Ruminococcaceae NA* | 4.8 ± 4.1 | 8.3 ± 6.4^a^ | 2.7 ± 6.7^a^ | 4.8 ± 5.3 | 4.9 ± 3.5 | 0.69 ± 4.4 | 3.7 ± 2.2 | 12.4 ± 10.1^b^ | 7.8 ± 10.7^b^ | 2.8 ± 3.2 | 1.8 ± 5.4^a,b^ | -0.8 ± 9.5^a,b^ |
| **Firmicutes** | *Oscillibacter* | 0.11 ± 0.19 | 0.28 ± 0.29^b^ | 0.1 ± 0.4 | 0.18 ± 0.3 | 0.08 ± 0.16^a^ | -0.11 ± 0.4 | 0.07 ± 0.15 | 0.35 ± 0.43^a,c^ | 0.27 ± 0.45^a^ | 0.06 ± 0.16 | 0.0 ± 0.03^b,c^ | -0.06 ± 0.15^a^ |
| **Firmicutes** | *Pseudoflavonifractor* | 0.06 ± 0.04 | 0.06 ± 0.06 | 0.0 ± 0.08 | 0.06 ± 0.05 | 0.06 ± 0.05 | -0.0 ± 0.07 | 0.05 ± 0.07 | 0.10 ± 0.07 | 0.05 ± 0.11 | 0.03 ± 0.03 | 0.06 ± 0.06 | 0.02 ± 0.08 |
| **Firmicutes** | *Ruminococcus* | 5.4 ± 2.2 | 6.4 ± 2.5 | 1.0 ± 3.8 | 4.6 ± 1.7 | 5.3 ± 2.5 | 0.69 ± 3.3 | 4.9 ± 2.8 | 4.6 ± 2.3 | -0.35 ± 3.4 | 4.0 ± 2.5 | 4.7 ± 7.5 | 0.63 ± 7.8 |
| **Firmicutes** | *Clostridia NA_NA_NA* | 5.6 ± 6.6 | 3.7 ± 1.8^a^ | -0.86 ± 5.2^a^ | 5.2 ± 5.1 | 4.1 ± 3.7^a^ | -2.8 ± 7.8 | 3.8 ± 2.3 | 2.3 ± 3.7 | -2.9 ± 4.0 | 2.9 ± 3.1 | 1.2 ± 1.6^a,a^ | -1.8 ± 4.1^a^ |
| **Firmicutes** | *Clostridium XVIII* | 0 | 0^c^ | 0^c^ | 0 | 0.0 ± 0.0^b^ | 0.0 ± 0.0^b^ | 0 | 0^c^ | 0^c^ | 0 | 0.1 ± 0.41^b,c,c^ | 0.1 ± 0.41^b,c,c^ |
| **Firmicutes** | *Erysipelotrichaceae NA* | 0.0 ± 0.01 | 0.0 ± 0.0 | 0.0 ± 0.0 | 0 | 0.0 ± 0.0 | 0.0 ± 0.0 | 0 | 0^a^ | 0 | 0 | 0.01 ± 0.14^a^ | 0.01 ± 0.14 |
| **Firmicutes** | *Turicibacter* | 0.71 ± 1.3^a^ | 0.06 ± 0.37 | -0.24 ± 0.99 | 0.8 ± 1.3^a^ | 0.07 ± 0.58 | -0.42 ± 1.3 | 3.1 ± 5.4^a,a^ | 0.17 ± 0.4 | -2.8 ± 5.5 | 5.1 ± 5.0 | 0.34 ± 2.9 | -2.1 ± 4.7 |
| **Firmicutes** | *Firmicutes NA_NA_NA_NA* | 2.2 ± 1.8 | 1.4 ± 1.2^b^ | -1.2 ± 1.6 | 1.9 ± 2.1 | 0.4 ± 0.9 | -1.2 ± 2.3 | 1.2 ± 0.4 | 0.9 ± 0.6 | -0.36 ± 1.0 | 1.4 ± 1.0 | 0.18 ± 0.5^b^ | -1.3 ± 1.2 |
| **Firmicutes** | *Dialister* | 0.0 ± 0.0 | 0.0 ± 0.0 | 0.0 ± 0.0 | 0.01 ± 0.02 | 0.0 ± 0.0 | -0.01 ± 0.02 | 0 | 0 | 0 | 0 | 0.0 ± 0.0 | 0.0 ± 0.0 |

a = statistical difference between groups at the corresponding column and row (p < 0.05)

b = statistical difference between groups at the corresponding column and row (p < 0.01)

c = statistical difference between groups at the corresponding column and row (p < 0.001)

NA = uncertain genera

**Table S1** continues…

|  |  | **Control** | | | **5-Fluorouracil** | | | **Oxaliplatin** | | | **Irinotecan** | | |
| --- | --- | --- | --- | --- | --- | --- | --- | --- | --- | --- | --- | --- | --- |
| **Phyla** | **Taxon** | **t1** | **t2** | **Δ** | **t1** | **t2** | **Δ** | **t1** | **t2** | **Δ** | **t1** | **t2** | **Δ** |
| **Fusobacteria** | *Fusobacterium* | 0.0 ± 0.0 | 0.01 ± 0.06^b^ | 0.01 ± 0.06^b^ | 0.0 ± 0.0 | 0.01 ± 0.04^b^ | 0.0 ± 0.03^b^ | 0 | 0.08 ± 5.4 | 0.08 ± 5.4 | 0 | 13.8 ± 16.0^b,b^ | 13.8 ± 16.0^b,b^ |
| **Proteobacteria** | *Gemmiger* | 0.01 ± 0.01 | 0.02 ± 0.04 | 0.01 ± 0.04 | 0.01 ± 0.03 | 0.02 ± 0.04 | 0.0 ± 0.05 | 0.01 ± 0.01 | 0.05 ± 0.13 | 0.04 ± 0.13 | 0.01 ± 0.01 | 0.01 ± 0.01 | -0.0 ± 0.01 |
| **Proteobacteria** | *Sphingomonas* | 0.04 ± 0.06 | 0.27 ± 0.32 | 0.23 ± 0.32 | 0.09 ± 0.15 | 0.85 ± 2.2 | 0.76 ± 2.3 | 0.04 ± 0.04 | 1.6 ± 3.9 | 1.5 ± 3.9 | 0.04 ± 0.04 | 0.33 ± 0.86 | 0.28 ± 0.87 |
| **Proteobacteria** | *Burkholderia* | 0.01 ± 0.03 | 0.01 ± 0.02 | -0.0 ± 0.03 | 0.06 ± 0.14 | 0.04 ± 0.06 | -0.02 ± 0.11 | 0.01 ± 0.01 | 0.17 ± 0.53 | 0.16 ± 0.53 | 0.01 ± 0.01 | 0.15 ± 0.46 | 0.15 ± 0.47 |
| **Proteobacteria** | *Variovorax* | 0.01 ± 0.02 | 0.01 ± 0.01 | 0.0 ± 0.03 | 0.26 ± 0.77 | 0.0 ± 0.01 | -0.26 ± 0.77 | 0.01 ± 0.01 | 0.06 ± 0.16 | 0.05 ± 0.16 | 0.01 ± 0.03 | 0.0 ± 0.0 | -0.01 ± 0.03 |
| **Proteobacteria** | *Parasutterella* | 0.24 ± 0.11 | 0.17 ± 0.1 | -0.05 ± 0.19^a^ | 0.16 ± 0.21 | 1.1 ± 1.3^a^ | 0.92 ± 1.2 | 0.2 ± 0.26 | 1.7 ± 6.7 | 1.5 ± 6.4^a,b^ | 0.31 ± 0.23 | 0.2 ± 0.4^a^ | -0.1 ± 0.66^b^ |
| **Proteobacteria** | *Desulfovibrio* | 0.06 ± 0.03 | 0.12 ± 0.04^a^ | 0.07 ± 0.11 | 0.05 ± 0.09 | 0.23 ± 0.25 | 0.15 ± 0.33 | 0.05 ± 0.06 | 0.59 ± 0.72^a,b^ | 0.56 ± 0.64^a^ | 0.05 ± 0.06 | 0.07 ± 0.12^b^ | 0.02 ± 0.03^a^ |
| **Proteobacteria** | *Desulfovibrionaceae NA* | 0.01 ± 0.01 | 0.04 ± 0.03^b,c^ | 0.04 ± 0.02^b^ | 0.01 ± 0.01 | 0.13 ± 0.18^a^ | 0.13 ± 0.18^b^ | 0.01 ± 0.01 | 0.42 ± 1.0^b^ | 0.41 ± 1.0^b^ | 0.01 ± 0.01 | 1.6 ± 5.2^a,c^ | 1.6 ± 5.2^b,b,b^ |
| **Proteobacteria** | *Desulfovibrionales NA_NA* | 0.01 ± 0.01 | 0.02 ± 0.14 | 0.01 ± 0.12 | 0.0 ± 0.01 | 0.04 ± 0.05 | 0.04 ± 0.04 | 0.0 ± 0.02 | 0.1 ± 0.24^b^ | 0.1 ± 0.21^a^ | 0.01 ± 0.01 | 0.01 ± 0.06^b^ | 0.0 ± 0.07^a^ |
| **Proteobacteria** | *Deltaproteobacteria NA_NA_NA* | 0.04 ± 0.14 | 0.13 ± 0.21^b^ | 0.07 ± 0.36^a^ | 0.07 ± 0.30 | 0.03 ± 0.05 | -0.06 ± 0.32 | 0.04 ± 0.13 | 0.03 ± 0.10 | -0.02 ± 0.09 | 0.06 ± 0.11 | 0.01 ± 0.03^b^ | -0.05 ± 0.15^a^ |
| **Proteobacteria** | *Escherichia/Shigella* | 0.01 ± 0.01 | 0.04 ± 0.08^b^ | 0.03 ± 0.08^a^ | 0.02 ± 0.01 | 1.6 ± 6.6 | 1.6 ± 6.7 | 0.02 ± 0.02 | 0.11 ± 10.4 | 0.1 ± 10.4 | 0.01 ± 0.02 | 8.4 ± 30.0^b^ | 8.4 ± 30.0^a^ |
| **Proteobacteria** | *Morganella* | 0 | 0.0 ± 0.0^b^ | 0.0 ± 0.0 | 0 | 0.0 ± 0.36^a^ | 0.0 ± 0.36 | 0 | 0.02 ± 0.59 | 0.02 ± 0.59 | 0 | 0.63 ± 2.8^a,b^ | 0.63 ± 2.8 |
| **Proteobacteria** | *Proteus* | 0.0 ± 0.0 | 0.0 ± 0.01^a^ | 0.0 ± 0.0 | 0 | 0.02 ± 0.48 | 0.02 ± 0.48 | 0.0 ± 0.0 | 0.05 ± 0.19 | 0.04 ± 0.19 | 0 | 0.09 ± 0.27^a^ | 0.09 ± 0.27 |
| **Synergistetes** | *Synergistia NA_NA_NA* | 0.55 ± 0.50 | 1.0 ± 0.73^b^ | 0.41 ± 0.73^b^ | 0.39 ± 0.55 | 1.0 ± 0.35^b^ | 0.53 ± 0.80^a^ | 0.38 ± 0.35 | 0.42 ± 1.0 | 0.16 ± 1.2 | 0.74 ± 0.66 | 0.12 ± 0.18^b,b^ | -0.64 ± 0.76^a,b^ |
| **Verrucomicrobia** | *Akkermansia* | 0.01 ± 0.04 | 0.4 ± 0.43^a^ | 0.37 ± 0.32 | 0.01 ± 0.22 | 1.9 ± 2.2^a^ | 1.9 ± 2.0 | 0.21 ± 0.50 | 1.1 ± 2.9 | 0.79 ± 3.1 | 0.12 ± 0.59 | 0.5 ± 0.94 | 0.09 ± 1.2 |

a = statistical difference between groups at the corresponding column and row (p < 0.05)

b = statistical difference between groups at the corresponding column and row (p < 0.01)

c = statistical difference between groups at the corresponding column and row (p < 0.001)

NA = uncertain genera

**Table S2** All identified serum metabolites with their respective chemical shift regions. Values are expressed as resonances at baseline (t1) and at end of the experiment (t2). All data are listed as mean ± standard deviation.

|  | |  | **Control** | | | **5-Fluorouracil** | | | **Oxaliplatin** | | | **Irinotecan** | | |
| --- | --- | --- | --- | --- | --- | --- | --- | --- | --- | --- | --- | --- | --- | --- |
| **ppm** | | **Metabolite** | **t1** | **t2** | **Δ** | **t1** | **t2** | **Δ** | **t1** | **t2** | **Δ** | **t1** | **t2** | **Δ** |
| 0.6 | 0.7 | **Choresterol** | 18.5 ± 1.1 | 21.0 ± 1.2^a^ | 2.5 ± 1.2^a^ | 18.8 ± 1.1 | 19.5 ± 1.1^a,c^ | 0.57 ± 1.1^a,c^ | 18.8 ± 0.95 | 20.3 ± 1.2 | 1.5 ± 1.4 | 19.0 ± 1.1 | 21.7 ± 1.0^c^ | 2.8 ± 1.8^b^ |
| 0.8 | 0.9 | **-CH_3_** | 58.7 ± 3.2 | 65.7 ± 2.3^b,c,c^ | 7.0 ± 4.3^a,c^ | 59.6 ± 2.9 | 71.4 ± 3.3^a,c^ | 12.0 ± 3.6^a^ | 59.5 ± 2.8 | 69.2 ± 2.1^b,c^ | 9.3 ± 4.1^b^ | 58.7 ± 2.8 | 74.9 ± 1.9^a,c,c^ | 16.2 ± 3.1^b,c^ |
| 0.95 | 0.97 | **Leucine** | 8.2 ± 0.59 | 9.6 ± 0.33^b,c^ | 1.4 ± 0.43^b^ | 8.3 ± 0.45 | 8.9 ± 0.33^c,c,c^ | 0.59 ± 0.32^a,b,c^ | 8.4 ± 0.54 | 9.6 ± 0.29^b,c^ | 1.2 ± 0.47^a,a^ | 8.3 ± 0.45 | 10.2 ± 0.31^b,b,c^ | 1.8 ± 0.59^a,c^ |
| 0.99 | 1.01 | **Isoleucine** | 6.3 ± 0.39 | 7.2 ± 0.36 | 0.92 ± 0.28^a^ | 6.3 ± 0.31 | 6.8 ± 0.39^b^ | 0.56 ± 0.25^a,b^ | 6.5 ± 0.43 | 7.1 ± 0.37 | 0.63 ± 0.29^a^ | 6.4 ± 0.33 | 7.4 ± 0.23^b^ | 1.0 ± 0.35^a,b^ |
| 1.02 | 1.05 | **Valine** | 7.9 ± 0.5 | 9.2 ± 0.38^c^ | 1.3 ± 0.42^c^ | 8.0 ± 0.39 | 8.3 ± 0.37^a,c,c^ | 0.27 ± 0.32^c,c^ | 8.0 ± 0.45 | 8.8 ± 0.33^a,a^ | 0.79 ± 0.41 | 8.0 ± 0.42 | 9.3 ± 0.36^a,c^ | 1.2 ± 0.62^c^ |
| 1.05 | 1.08 | **Methylsuccinate** | 8.0 ± 0.48 | 9.0 ± 0.32^b,b^ | 1.0 ± 0.45^b^ | 8.1 ± 0.44 | 8.4 ± 0.33^a,b,c^ | 0.26 ± 0.35^b,c^ | 8.1 ± 0.43 | 8.8 ± 0.29^a,c^ | 0.71 ± 0.45^a^ | 8.1 ± 0.42 | 9.5 ± 0.33^b,c,c^ | 1.4 ± 0.62^a,c^ |
| 1.18 | 1.2 | **3-hydroxybutyrate** | 6.6 ± 0.44 | 8.0 ± 0.39^c^ | 1.4 ± 0.60^a^ | 6.8 ± 0.42 | 8.1 ± 0.38^c^ | 1.3 ± 0.52^a^ | 6.7 ± 0.49 | 8.2 ± 0.39^b^ | 1.5 ± 0.62 | 6.7 ± 0.41 | 8.9 ± 0.3^b,c,c^ | 2.1 ± 0.55^a,a^ |
| 1.2 | 1.27 | **VLDL** | 54.0 ± 3.7 | 57.4 ± 7.0^b,c,c^ | 3.4 ± 8.1^a,c,c^ | 55.2 ± 5.6 | 78.7 ± 8.5^b,c^ | 23.9 ± 8.7^b,c^ | 53.3 ± 3.2 | 67.0 ± 6.1^b,b^ | 12.9 ± 6.7^a,b^ | 52.6 ± 2.8 | 73.5 ± 4.4^c^ | 21.2 ± 3.8^c^ |
| 1.27 | 1.3 | **LDL** | 25.7 ± 4.1 | 29.1 ± 6.3^c^ | 3.4 ± 5.3^c^ | 25.0 ± 5.9 | 42.7 ± 8.0^c,c,c^ | 18.7 ± 7.8^c,c,c^ | 25.4 ± 2.6 | 29.3 ± 4.3^c^ | 4.0 ± 2.4^c^ | 23.1 ± 3.2 | 28.7 ± 4.5^c^ | 6.2 ± 3.9^c^ |
| 1.31 | 1.34 | **Lactate** | 20.9 ± 1.6 | 28.5 ± 2.2^a,c^ | 7.7 ± 1.7^a,c^ | 22.8 ± 5.5 | 35.0 ± 2.6^b,c^ | 13.8 ± 3.0^c^ | 20.8 ± 1.2 | 32.5 ± 3.6^a^ | 11.4 ± 2.9^a^ | 20.0 ± 1.8 | 30.2 ± 3.7^b^ | 10.4 ± 4.0 |
| 1.36 | 1.38 | **Acetoin** | 5.6 ± 0.30 | 6.1 ± 0.30^a,b^ | 0.57 ± 0.27^a^ | 5.6 ± 0.21 | 5.8 ± 0.34^a^ | 0.28 ± 0.39 | 5.6 ± 0.22 | 5.7 ± 0.26^b^ | 0.11 ± 0.31^a^ | 5.5 ± 0.22 | 5.9 ± 0.24 | 0.33 ± 0.39 |
| 1.46 | 1.48 | **Alanine** | 6.5 ± 0.43 | 7.8 ± 0.27^c,c,c^ | 1.2 ± 0.53^a,b^ | 6.8 ± 0.44 | 7.1 ± 0.29^c^ | 0.30 ± 0.51^b^ | 6.5 ± 0.28 | 7.2 ± 0.25^c^ | 0.71 ± 0.50 | 6.5 ± 0.36 | 7.2 ± 0.20^c^ | 0.63 ± 0.42^a^ |
| 1.5 | 1.64 | **B-CH_2_** | 45.2 ± 0.43 | 43.1 ± 0.64^b,c^ | -2.1 ± 0.62^a,a,c^ | 44.8 ± 0.86 | 43.7 ± 0.79^c,c^ | -1.3 ± 0.74^a,c,c^ | 45.1 ± 0.27 | 41.6 ± 0.66^c,c^ | -3.4 ± 0.65^c,c^ | 45.0 ± 0.39 | 42.1 ± 0.67^b,c^ | -2.9 ± 0.69^a,c^ |
| 1.64 | 1.74 | **Arginine** | 33.0 ± 1.5 | 28.6 ± 1.3^c,c,c^ | -4.3 ± 1.9^b,c,c^ | 32.4 ± 2.3 | 24.0 ± 1.5^c^ | -8.9 ± 1.8^c^ | 32.5 ± 1.1 | 25.2 ± 0.64^c^ | -7.2 ± 1.2^b^ | 33.1 ± 1.3 | 24.4 ± 0.74^c^ | -8.7 ± 1.3^c^ |
| 1.9 | 1.91 | **Acetate** | 2.5 ± 0.19 | 2.5 ± 0.21^a,b,b^ | 0.0 ± 0.11^c,c,c^ | 2.6 ± 0.18 | 2.2 ± 0.20^a^ | -0.39 ± 0.19^c^ | 2.4 ± 0.11 | 2.2 ± 0.14^b^ | -0.27 ± 0.10^c^ | 2.5 ± 0.11 | 2.2 ± 0.13^b^ | -0.35 ± 0.08^c^ |
| 1.96 | 2.03 | **CH_2_-CH=C** | 37.8 ± 3.1 | 30.4 ± 1.7^c^ | -7.4 ± 2.6 | 37.5 ± 2.4 | 33.2 ± 1.5^a,c,c^ | -4.9 ± 2.0 | 37.0 ± 2.4 | 30.4 ± 1.3^c^ | -6.4 ± 1.2 | 37.5 ± 2.1 | 31.5 ± 0.75^a^ | -6.0 ± 2.3 |
| 2.03 | 2.05 | **NAC1** | 19.4 ± 1.2 | 15.4 ± 0.55^a,b^ | -4.0 ± 1.2 | 18.5 ± 1.3 | 14.8 ± 0.64^a^ | -4.0 ± 0.80 | 19.4 ± 0.84 | 14.9 ± 0.46 | -4.4 ± 1.1 | 19.3 ± 0.80 | 14.6 ± 0.31^b^ | -4.5 ± 0.88 |
| 2.05 | 2.09 | **NAC2** | 20.4 ± 1.2^a^ | 16.6 ± 1.6^a,c,c^ | -3.8 ± 1.6^a,a,b^ | 19.1 ± 1.2^a,a^ | 13.9 ± 0.86^c^ | -5.4 ± 0.90^a^ | 20.7 ± 1.1^a^ | 15.2 ± 1.0^a^ | -5.4 ± 1.2^a^ | 20.1 ± 0.89 | 14.5 ± 0.32^c^ | -5.5 ± 0.88^b^ |
| 2.10 | 2.16 | **Glutamine** | 18.0 ± 0.76 | 20.3 ± 0.84^a,b,c^ | 2.3 ± 1.2^a,c^ | 18.5 ± 0.88 | 17.7 ± 1.0^b,b,c^ | -0.89 ± 1.2^a,b,c^ | 18.1 ± 0.68 | 19.0 ± 0.53^b,b^ | 0.83 ± 0.91^a,a^ | 18.2 ± 0.81 | 19.1 ± 0.80^a,b^ | 0.94 ± 1.4^b^ |
| 2.20 | 2.24 | **a-CH2** | 10.7 ± 0.62 | 10.6 ± 1.2^c^ | -0.11 ± 0.97^c^ | 10.8 ± 0.97 | 13.2 ± 1.4^c,c,c^ | 2.4 ± 1.3^c,c,c^ | 10.5 ± 0.62 | 10.2 ± 0.76^c^ | -0.25 ± 0.32^c^ | 10.4 ± 0.63 | 9.7 ± 0.71^c^ | -0.58 ± 0.85^c^ |
| 2.265 | 2.28 | **Acetoacetate** | 5.6 ± 0.32 | 4.2 ± 0.11^c^ | -1.4 ± 0.33 | 5.4 ± 0.41 | 3.9 ± 0.2^a,b,c^ | -1.6 ± 0.26 | 5.6 ± 0.25 | 4.1 ± 0.13^a^ | -1.5 ± 0.26 | 5.6 ± 0.27 | 4.1 ± 0.10^b^ | -1.5 ± 0.28 |
| 2.32 | 2.36 | **Glutamate** | 15.5 ± 1.9 | 7.8 ± 0.45^a,a,c^ | -7.6 ± 2.1 | 14.4 ± 2.0 | 7.2 ± 0.62^a^ | -7.6 ± 1.2 | 15.2 ± 1.4 | 7.2 ± 0.38^a^ | -7.8 ± 1.4 | 15.3 ± 1.6 | 6.9 ± 0.26^c^ | -8.4 ± 1.6 |
| 2.36 | 2.37 | **Pyruvate** | 2.3 ± 0.12 | 1.9 ± 0.12^b,b,c^ | -0.44 ± 0.18^a,b^ | 2.2 ± 0.08 | 1.7 ± 0.14^b^ | -0.49 ± 0.11 | 2.4 ± 0.12 | 1.8 ± 0.07^b^ | -0.61 ± 0.13^a^ | 2.3 ± 0.1 | 1.7 ± 0.06^c^ | -0.63 ± 0.09^b^ |
| 2.53 | 2.55 | **Citrate** | 1.4 ± 0.09 | 1.4 ± 0.08^c,c,c^ | 0.02 ± 0.11^c,c,c^ | 1.4 ± 0.11 | 1.1 ± 0.15^c^ | -0.31 ± 0.13^c^ | 1.4 ± 0.08 | 1.1 ± 0.06^c^ | -0.24 ± 0.10^c^ | 1.4 ± 0.11 | 1.1 ± 0.06^c^ | -0.38 ± 0.14^c^ |
| 2.598 | 2.602 | **Methylamine** | 0.53 ± 0.03 | 0.55 ± 0.03^c,c,c^ | 0.02 ± 0.04^b,c,c^ | 0.53 ± 0.04 | 0.44 ± 0.05^c^ | -0.10 ± 0.05^c^ | 0.52 ± 0.03 | 0.46 ± 0.02^c^ | -0.06 ± 0.03^b^ | 0.53 ± 0.04 | 0.44 ± 0.03^c^ | -0.09 ± 0.06^c^ |
| 2.605 | 2.63 | **Methylsuccinate** | 1.6 ± 0.09 | 1.7 ± 0.08^c,c,c^ | 0.10 ± 0.12^b,c,c^ | 1.7 ± 0.11 | 1.4 ± 0.12^c^ | -0.29 ± 0.14^c^ | 1.6 ± 0.07 | 1.5 ± 0.04^c^ | -0.14 ± 0.08^b^ | 1.6 ± 0.11 | 1.4 ± 0.08^c^ | -0.22 ± 0.15^c^ |
| 2.63 | 2.65 | **Methionine** | 1.2 ± 0.06 | 1.3 ± 0.06^c,c,c^ | 0.08 ± 0.08^c,c,c^ | 1.2 ± 0.08 | 1.1 ± 0.08^c^ | -0.20 ± 0.09^a,c^ | 1.2 ± 0.04 | 1.1 ± 0.03^c^ | -0.09 ± 0.04^a,c^ | 1.2 ± 0.06 | 1.1 ± 0.05^c^ | -0.14 ± 0.09^c^ |

a = statistical difference between groups at the corresponding column and row (p < 0.05)

b = statistical difference between groups at the corresponding column and row (p < 0.01)

c = statistical difference between groups at the corresponding column and row (p < 0.001)

**Table S2** continues…

|  | |  | **Control** | | | **5-Fluorouracil** | | | **Oxaliplatin** | | | **Irinotecan** | | |
| --- | --- | --- | --- | --- | --- | --- | --- | --- | --- | --- | --- | --- | --- | --- |
| **ppm** | | **Metabolite** | **t1** | **t2** | **Δ** | **t1** | **t2** | **Δ** | **t1** | **t2** | **Δ** | **t1** | **t2** | **Δ** |
| 2.645 | 2.655 | **Malate** | 0.66 ± 0.04 | 0.71 ± 0.04^c,c,c^ | 0.05 ± 0.04^c,c,c^ | 0.68 ± 0.05 | 0.57 ± 0.04^c^ | -0.12 ± 0.05^a,c^ | 0.65 ± 0.02 | 0.60 ± 0.02^c^ | -0.05 ± 0.02^a,c^ | 0.66 ± 0.03 | 0.59 ± 0.03^c^ | -0.08 ± 0.05^c^ |
| 2.71 | 2.72 | **Dimethylamine** | 1.2 ± 0.04 | 1.3 ± 0.06^a^ | 0.09 ± 0.06 | 1.2 ± 0.05 | 1.4 ± 0.08^a,a^ | 0.14 ± 0.11 | 1.2 ± 0.04 | 1.3 ± 0.08^a^ | 0.07 ± 0.10 | 1.2 ± 0.05 | 1.3 ± 0.05 | 0.14 ± 0.06 |
| 2.72 | 2.82 | **=CH-CH_2_-CH=** | 15.6 ± 1.0 | 17.3 ± 1.4^c,c,c^ | 1.7 ± 1.5^c,c,c^ | 16.1 ± 1.4 | 21.5 ± 1.5^b,c^ | 5.6 ± 1.4^a,c^ | 16.1 ± 0.97 | 21.7 ± 0.64^b,c^ | 5.5 ± 1.3^a,c^ | 16.0 ± 1.1 | 23.5 ± 0.95^b,b,c^ | 7.6 ± 1.3^a,a,c^ |
| 2.85 | 3.02 | **Albumin lysil** | 15.0 ± 1.1 | 18.1 ± 0.85^c,c,c^ | 2.2 ± 1.1^c,c,c^ | 16.1 ± 0.93 | 14.5 ± 0.96^c^ | -1.7 ± 1.2^c^ | 15.7 ± 0.73 | 15.2 ± 0.42^c^ | -0.63 ± 0.71^c^ | 15.9 ± 0.76 | 14.6 ± 0.65^c^ | -1.4 ± 1.1^c^ |
| 3.02 | 3.025 | **Creatine** | 0.57 ± 0.04 | 0.68 ± 0.06^a,a,b^ | 0.11 ± 0.07^a,b^ | 0.60 ± 0.04 | 0.62 ± 0.04^a^ | 0.03 ± 0.06^a^ | 0.57 ± 0.04 | 0.62 ± 0.02^a^ | 0.06 ± 0.05 | 0.58 ± 0.05 | 0.60 ± 0.03^b^ | 0.02 ± 0.05^b^ |
| 3.03 | 3.032 | **Creatine-P** | 0.27 ± 0.04^a^ | 0.29 ± 0.05 | 0.03 ± 0.03 | 0.32 ± 0.03^a^ | 0.35 ± 1.0 | 0.02 ± 1.0 | 0.25 ± 0.03 | 0.29 ± 0.08 | 0.03 ± 0.06 | 0.29 ± 0.04 | 0.30 ± 0.08 | 0.01 ± 0.08 |
| 3.034 | 3.038 | **Creatinine** | 0.50 ± 0.10 | 0.69 ± 0.18 | 0.19 ± 0.20 | 0.55 ± 0.29 | 0.71 ± 0.15 | 0.26 ± 0.14 | 0.54 ± 0.10 | 0.75 ± 0.14 | 0.21 ± 0.13 | 0.52 ± 0.10 | 0.75 ± 0.15 | 0.24 ± 0.11 |
| 3.18 | 3.2 | **Choline** | 3.2 ± 0.54 | 3.8 ± 0.71^b,b^ | 0.62 ± 0.60^b,c^ | 3.4 ± 0.54 | 3.0 ± 0.45^b^ | -0.49 ± 0.62^c^ | 3.3 ± 0.42 | 3.0 ± 0.46^b^ | -0.26 ± 0.62^b^ | 3.5 ± 0.36 | 3.5 ± 0.50 | -0.04 ± 0.47 |
| 3.2 | 3.225 | **N(CH_3_)_3_** | 11.3 ± 0.60 | 9.2 ± 0.74^c,c,c^ | -2.1 ± 0.64^c,c,c^ | 10.9 ± 0.52 | 10.9 ± 0.69^c,c^ | -0.19 ± 0.60^c,c^ | 11.1 ± 0.77 | 11.2 ± 0.88^c,c^ | 0.01 ± 1.1^c,c^ | 11.1 ± 0.43 | 13.9 ± 0.57^c,c,c^ | 2.8 ± 0.81^c,c,c^ |
| 3.33 | 3.36 | **Proline** | 6.9 ± 1.1 | 2.3 ± 0.32^b^ | -4.6 ± 1.2 | 6.1 ± 1.2 | 2.1 ± 0.26 | -4.3 ± 0.69 | 6.9 ± 0.82 | 2.1 ± 0.24 | -4.7 ± 0.89 | 6.8 ± 1.0 | 2.0 ± 0.17^b^ | -4.8 ± 1.0 |
| 3.55 | 3.555 | **Glycine** | 1.0 ± 0.11 | 1.1 ± 0.20^b^ | 0.11 ± 0.25^a,b^ | 1.0 ± 0.10 | 0.87 ± 0.15^a,b^ | -0.16 ± 0.17^a^ | 1.1 ± 0.10 | 1.1 ± 0.23^a^ | -0.01 ± 0.22 | 1.1 ± 0.09 | 0.95 ± 0.08 | -0.18 ± 0.08^b^ |
| 3.575 | 3.59 | **Threonine** | 2.6 ± 0.16 | 2.2 ± 0.11^c,c,c^ | -0.45 ± 0.21^a,c,c^ | 2.5 ± 0.26 | 1.6 ± 0.13^a,c,c^ | -0.95 ± 0.18^a,c^ | 2.6 ± 0.11 | 1.9 ± 0.11^c,c^ | -0.71 ± 0.18^a,a^ | 2.6 ± 0.14 | 1.8 ± 0.07^a,c^ | -0.86 ± 0.15^c^ |
| 3.79 | 3.8 | **Nitrosodimethylamine** | 1.8 ± 0.05 | 1.7 ± 0.11^b,c,c^ | -0.04 ± 0.11^a,c,^*^c^* | 1.8 ± 0.07 | 1.4 ± 0.10^c,c^ | -0.38 ± 0.11^b,c^ | 1.8 ± 0.08 | 1.6 ± 0.10^a,b,c^ | -0.18 ± 0.13^a,b^ | 1.8 ± 0.06 | 1.5 ± 0.04^a,^*^c^* | -0.30 ± 0.06*^c^* |
| 3.81 | 3.86 | **Glucose** | 13.6 ± 0.66 | 17.3 ± 1.1^b^ | 3.6 ± 1.0^a^ | 14.2 ± 1.2 | 16.7 ± 1.2^c^ | 3.0 ± 1.4^b^ | 13.9 ± 0.44 | 19.2 ± 1.2^b,b,c^ | 5.3 ± 1.3^a,b,b^ | 14.1 ± 0.66 | 17.3 ± 1.2^b^ | 3.1 ± 1.4^b^ |
| 5.2 | 5.25 | **α-Glucose** | 3.4 ± 0.24 | 5.2 ± 0.36^a,b,c^ | 1.8 ± 0.42^c^ | 3.7 ± 0.79 | 5.8 ± 0.43^a,b^ | 2.3 ± 0.57 | 3.5 ± 0.22^a^ | 6.5 ± 0.41^b,b,c^ | 3.1 ± 0.53^a,a,c^ | 3.5 ± 0.32 | 5.9 ± 0.54^b,b^ | 2.3 ± 0.65^a^ |
| 6.87 | 6.9 | **Tyrosine** | 1.1 ± 0.10 | 1.2 ± 0.08^c,c,c^ | 0.05 ± 0.07^b,c,c^ | 1.2 ± 0.10^a^ | 1.0 ± 0.08^c^ | -0.16 ± 0.10^a,c^ | 1.0 ± 0.04^a^ | 0.98 ± 0.03^c^ | -0.06 ± 0.04^a,a,b^ | 1.1 ± 0.06 | 0.94 ± 0.05^c^ | -0.15 ± 0.06^a,c^ |
| 7.23 | 7.3 | **Tryptophan** | 3.4 ± 0.21 | 3.8 ± 0.12^c,c,c^ | 0.36 ± 0.25^c,c,c^ | 3.5 ± 0.21 | 3.1 ± 0.21^c^ | -0.40 ± 0.31^c^ | 3.4 ± 0.13 | 3.1 ± 0.09^a,c^ | -0.24 ± 0.16^c^ | 3.4 ± 0.16 | 2.9 ± 0.12^a,c^ | -0.45 ± 0.17^c^ |
| 7.3 | 7.45 | **Phenylalanine** | 6.2 ± 0.37 | 6.7 ± 0.31^c,c,c^ | 0.54 ± 0.39^c,c,c^ | 6.3 ± 0.39 | 5.7 ± 0.45^c^ | -0.58 ± 0.53^c^ | 5.9 ± 0.20 | 5.7 ± 0.17^c^ | -0.26 ± 0.29^c^ | 6.1 ± 0.26 | 5.4 ± 0.27^c^ | -0.67 ± 0.30^c^ |

a = statistical difference between groups at the corresponding column and row (p < 0.05)

b = statistical difference between groups at the corresponding column and row (p < 0.01)

c = statistical difference between groups at the corresponding column and row (p < 0.001)

**Table S3** All identified urine metabolites with their respective chemical shift regions. Values are expressed as resonances at baseline (t1) and at end of the experiment (t2). All data are listed as mean ± standard deviation.

|  | |  | **Control** | | | **5-Fluorouracil** | | | **Oxaliplatin** | | | **Irinotecan** | | |
| --- | --- | --- | --- | --- | --- | --- | --- | --- | --- | --- | --- | --- | --- | --- |
| **ppm** | | **Metabolite** | **t1** | **t2** | **Δ** | **t1** | **t2** | **Δ** | **t1** | **t2** | **Δ** | **t1** | **t2** | **Δ** |
| 0.81 | 0.83 | **2-hydroxybutyrate** | 1.0 ± 0.20 | 1.9 ± 0.37^a^ | 0.85 ± 0.29^a^ | 1.0 ± 0.20 | 1.7 ± 0.34 | 0.63 ± 0.28 | 0.9 ± 0.17 | 1.4 ± 0.23^a^ | 0.46 ± 0.20^a^ | 0.85 ± 0.19 | 1.6 ± 0.49 | 0.73 ± 0.42 |
| 0.83 | 0.88 | **CH_3_** | 6.0 ± 0.55 | 10.8 ± 1.9 | 4.8 ± 1.6 | 5.9 ± 0.54 | 9.5 ± 1.3 | 3.5 ± 1.2 | 5.7 ± 0.74 | 9.6 ± 1.4 | 3.8 ± 1.1 | 5.6 ± 1.3 | 9.0 ± 3.0 | 3.4 ± 3.1 |
| 1.1 | 1.3 | **(CH_2_)_n_** | 24.0 ± 1.7^a^ | 30.8 ± 3.0^a,c,c^ | 6.7 ± 2.8^a,b,c^ | 29.8 ± 8.4^a,a,b^ | 26.5 ± 2.8^a,b,c^ | -2.8 ± 8.0^c^ | 23.3 ± 1.8^a^ | 20.5 ± 2.3^c,c^ | -2.7 ± 2.2^b^ | 22.2 ± 3.7^b^ | 21.5 ± 3.9^b,c^ | -0.04 ± 4.7^a^ |
| 0.935 | 0.965 | **Leucine** | 2.5 ± 0.27 | 2.8 ± 0.38^c^ | 0.36 ± 0.43^c^ | 2.4 ± 0.23 | 2.8 ± 0.56^c^ | 0.38 ± 0.63^c^ | 2.3 ± 0.17 | 3.6 ± 1.6^c^ | 1.2 ± 1.6^c^ | 2.4 ± 0.51 | 7.8 ± 4.1^c,c,c^ | 5.4 ± 3.9^c,c,c^ |
| 0.99 | 1.01 | **Isoleucine** | 0.80 ± 0.23 | 1.0 ± 0.20^c^ | 0.20 ± 0.29^c^ | 0.89 ± 0.15 | 0.97 ± 0.26^c^ | 0.07 ± 0.26^c^ | 0.73 ± 0.04 | 0.76 ± 0.28^c^ | 0.04 ± 0.26^c^ | 0.80 ± 0.15 | 2.0 ± 0.70^c,c,c^ | 1.2 ± 0.63^c,c,c^ |
| 1.03 | 1.04 | **Propionate** | 0.58 ± 0.09 | 0.64 ± 0.10^b^ | 0.07 ± 0.11^c^ | 0.62 ± 0.16 | 0.57 ± 0.21^b^ | -0.07 ± 0.28^c^ | 0.51 ± 0.04 | 0.69 ± 0.46^b^ | 0.18 ± 0.45^b^ | 0.53 ± 0.10 | 1.5 ± 0.86^b,b,b^ | 0.99 ± 0.81^b,c,c^ |
| 1.04 | 1.05 | **Valine** | 0.60 ± 0.13 | 0.63 ± 0.10^b^ | 0.03 ± 0.13^b^ | 0.63 ± 0.12 | 0.60 ± 0.23^b^ | -0.04 ± 0.28^b^ | 0.52 ± 0.04 | 0.81 ± 0.63^b^ | 0.29 ± 0.62^a^ | 0.55 ± 0.11 | 1.5 ± 0.92^b,b,b^ | 0.99 ± 0.86^a,b,b^ |
| 1.16 | 1.19 | **Ethanol** | 3.9 ± 0.28 | 4.5 ± 0.35^b,c^ | 0.59 ± 0.27^a,b^ | 4.6 ± 1.0^a,b^ | 4.0 ± 0.81^b^ | -0.68 ± 1.4^a^ | 3.8 ± 0.51^a^ | 3.1 ± 0.39^b,c^ | -0.73 ± 0.36^b^ | 3.6 ± 0.61^b^ | 3.4 ± 0.81^b^ | -0.07 ± 0.90 |
| 1.23 | 1.25 | **Palmitic/Dodecanoate** | 3.4 ± 0.20 | 4.1 ± 0.35^a,c,c^ | 0.70 ± 0.34^a,b,b^ | 3.5 ± 0.23 | 3.7 ± 0.28^a,b^ | 0.15 ± 0.20^a^ | 3.5 ± 0.37 | 3.4 ± 0.30^c^ | -0.08 ± 0.36^b^ | 3.2 ± 0.54 | 3.0 ± 0.41^b,c^ | -0.01 ± 0.76^b^ |
| 1.27 | 1.3 | **Valerate** | 4.2 ± 0.41 | 6.4 ± 1.0^b,c,c^ | 2.2 ± 0.89^c,c,c^ | 4.3 ± 0.37 | 5.1 ± 0.71^b,b,b^ | 0.73 ± 0.70^a,c^ | 4.1 ± 0.30 | 3.7 ± 0.57^b,c^ | -0.40 ± 0.49^a,c^ | 4.0 ± 0.71 | 3.6 ± 0.86^b,c^ | -0.20 ± 1.0^c^ |
| 1.35 | 1.36 | **3-aminoisovalerate** | 1.2 ± 0.14 | 1.3 ± 0.12^c^ | 0.12 ± 0.15^c^ | 1.3 ± 0.26 | 1.3 ± 0.21^c^ | 0.01 ± 0.25^b^ | 1.2 ± 0.10 | 0.79 ± 0.12^c,c,c^ | -0.38 ± 0.13^b,b,c^ | 1.1 ± 0.30 | 1.2 ± 0.20^c^ | 0.08 ± 0.36^b^ |
| 1.46 | 1.49 | **Alanine** | 3.8 ± 0.21 | 4.4 ± 0.45 | 0.58 ± 0.35^a^ | 3.8 ± 0.23 | 4.0 ± 0.45 | 0.19 ± 0.33^b^ | 3.6 ± 0.24 | 3.5 ± 0.72^b^ | -0.12 ± 0.69^c^ | 3.4 ± 0.59 | 5.1 ± 1.9^b^ | 1.8 ± 1.7^a,b,c^ |
| 1.54 | 1.6 | **B-CH_2_** | 8.5 ± 0.66 | 10.6 ± 1.2^a,b,c^ | 2.1 ± 1.2^a,b,c^ | 8.4 ± 0.49 | 8.8 ± 1.1^a^ | 0.35 ± 0.96^b^ | 8.3 ± 0.49 | 7.4 ± 0.83^c^ | -0.87 ± 0.69^c^ | 8.0 ± 1.4 | 8.2 ± 1.7^b^ | 0.41 ± 1.7^a^ |
| 1.74 | 1.78 | **Putrescine** | 6.9 ± 0.38 | 6.7 ± 0.58^b^ | -0.15 ± 0.45 | 6.6 ± 0.58 | 6.0 ± 0.80^a^ | -0.67 ± 0.69^a^ | 6.7 ± 0.33 | 5.6 ± 0.52^b,b^ | -1.2 ± 0.64^b^ | 6.5 ± 1.0 | 7.0 ± 0.97^a,b^ | 0.61 ± 1.6^a,b^ |
| 1.88 | 1.91 | **Arginine** | 4.1 ± 0.29 | 4.2 ± 0.43 | 0.02 ± 0.31 | 4.0 ± 0.34 | 5.4 ± 3.6 | 1.4 ± 3.6 | 3.9 ± 0.28 | 5.2 ± 2.2 | 1.3 ± 2.2 | 4.0 ± 0.56 | 5.7 ± 2.2 | 1.7 ± 2.3 |
| 1.91 | 1.93 | **Acetate** | 4.0 ± 0.49 | 3.8 ± 0.34^c^ | -0.28 ± 0.58^b^ | 4.5 ± 0.80 | 6.4 ± 4.3^c^ | 1.9 ± 4.1^a^ | 4.2 ± 1.0 | 9.7 ± 6.0^b^ | 5.4 ± 6.0 | 6.2 ± 8.4 | 23.4 ± 14.6^b,c,c^ | 16.3 ± 18.9^a,b^ |
| 1.98 | 2.01 | **2-hydroxyglutarate** | 8.4 ± 0.44 | 8.8 ± 0.73^a,b^ | 0.39 ± 0.70 | 8.0 ± 0.66 | 7.1 ± 1.0^a^ | -0.98 ± 1.1 | 8.3 ± 0.43 | 6.9 ± 1.6^b^ | -1.4 ± 1.5 | 7.7 ± 1.6 | 7.9 ± 1.5 | 0.27 ± 2.5 |
| 2.02 | 2.08 | **N-acetylglycoproteins** | 25.5 ± 0.68 | 23.3 ± 0.86^b^ | -2.2 ± 0.94 | 24.5 ± 1.0 | 21.9 ± 1.7 | -2.6 ± 2.1 | 25.3 ± 0.67 | 23.1 ± 2.2^b^ | -2.1 ± 1.7 | 24.6 ± 4.7 | 19.6 ± 3.2^b,b^ | -4.4 ± 6.0 |
| 2.18 | 2.2 | **Acetoin** | 4.8 ± 0.29 | 5.7 ± 0.45^b,c,c^ | 0.85 ± 0.44^a,c,c^ | 4.6 ± 0.89 | 4.7 ± 0.89^b,c^ | -0.06 ± 0.83^a,a^ | 4.8 ± 0.21 | 4.1 ± 0.62^a,c^ | -0.71 ± 0.70^c^ | 4.6 ± 0.87 | 3.3 ± 0.31^a,c,c^ | -1.2 ± 1.0^a,c^ |
| 2.27 | 2.28 | **Acetoacetate** | 2.2 ± 0.42 | 2.0 ± 0.23^a^ | -0.29 ± 0.37 | 2.8 ± 1.1 | 3.0 ± 1.5^a,b^ | 0.19 ± 1.1 | 2.2 ± 0.46 | 2.0 ± 0.73 | -0.27 ± 0.82 | 2.3 ± 0.61 | 1.3 ± 0.31^b^ | -0.80 ± 0.62 |
| 2.36 | 2.38 | **Pyruvate** | 3.7 ± 0.36 | 4.0 ± 0.29^c,c,c^ | 0.23 ± 0.22^b,c^ | 3.6 ± 0.42 | 3.2 ± 0.38^c^ | -0.53 ± 0.47^b^ | 3.8 ± 0.30 | 2.8 ± 0.36^c^ | -0.93 ± 0.41^b,c^ | 3.4 ± 0.40 | 3.1 ± 0.44^c^ | -0.19 ± 0.62^b^ |
| 2.39 | 2.42 | **Succinate** | 18.8 ± 3.3 | 19.5 ± 2.0^c^ | 0.47 ± 2.6^a,b^ | 16.9 ± 7.4^a^ | 7.5 ± 6.3^a,c^ | -10.6 ± 8.1^b^ | 23.0 ± 3.3^a^ | 13.5 ± 5.8 | -8.8 ± 6.2^a^ | 20.8 ± 2.0 | 15.7 ± 9.6^a^ | -5.2 ± 10.6 |
| 2.42 | 2.46 | **2-oxoglutarate** | 39.5 ± 4.3 | 32.1 ± 6.0^c,c,c^ | -7.1 ± 4.3^c,c,c^ | 40.0 ± 8.0 | 12.8 ± 4.1^c^ | -28.3 ± 7.2^c^ | 42.1 ± 7.9 | 10.6 ± 4.0^c^ | -32.2 ± 7.8^c^ | 43.7 ± 10.1 | 15.4 ± 10.0^c^ | -27.9 ± 15.7^c^ |
| 2.52 | 2.57 | **Citrate** | 35.1 ± 3.8^a^ | 26.4 ± 2.6^c,c,c^ | -8.8 ± 3.0^c,c,c^ | 42.4 ± 14.2 | 9.0 ± 3.2^c,c^ | -36.0 ± 11.1^c^ | 34.2 ± 2.7^a^ | 6.2 ± 3.4^c^ | -27.4 ± 4.1^b,c^ | 46.8 ± 10.6^a,a^ | 3.3 ± 0.67^c,c^ | -42.2 ± 11.8^b,c^ |
| 2.6 | 2.61 | **Methylamine** | 1.4 ± 0.36 | 1.3 ± 0.27 | -0.11 ± 0.33 | 3.7 ± 5.1 | 1.8 ± 1.1^b,b^ | -0.87 ± 4.1 | 1.4 ± 0.27 | 0.64 ± 0.34^b^ | -0.81 ± 0.54 | 1.6 ± 0.53 | 0.57 ± 0.22^b^ | -1.0 ± 0.74 |
| 2.62 | 2.65 | **Methionine** | 3.5 ± 0.45 | 4.1 ± 0.68^a,c,c^ | 0.57 ± 0.66^a,c,c^ | 3.8 ± 0.40 | 3.0 ± 1.4^a,b,b^ | -0.77 ± 1.2^a,a,a^ | 3.7 ± 0.42 | 1.5 ± 0.91^b,c^ | -2.2 ± 0.83^a,c^ | 3.4 ± 0.66 | 1.3 ± 0.36^b,c^ | -2.1 ± 0.93^a,c^ |
| 2.7 | 2.72 | **Dimethylamine** | 29.7 ± 3.6^c,c^ | 18.4 ± 3.9^c,c,c^ | -11.5 ± 2.3^c^ | 16.9 ± 3.2^c,c^ | 3.9 ± 1.5^c^ | -13.2 ± 3.4^c^ | 29.2 ± 3.1^c,c^ | 6.3 ± 1.7^c,c^ | -22.6 ± 3.2^c,c,c^ | 16.9 ± 4.0^c,c^ | 1.5 ± 0.35^c,c^ | -15.3 ± 4.7^c^ |

a = statistical difference between groups at the corresponding column and row (p < 0.05)

b = statistical difference between groups at the corresponding column and row (p < 0.01)

c = statistical difference between groups at the corresponding column and row (p < 0.001)

**Table S3** continues…

|  | |  | **Control** | | | **5-Fluorouracil** | | | **Oxaliplatin** | | | **Irinotecan** | | |
| --- | --- | --- | --- | --- | --- | --- | --- | --- | --- | --- | --- | --- | --- | --- |
| **ppm** | | **Metabolite** | **t1** | **t2** | **Δ** | **t1** | **t2** | **Δ** | **t1** | **t2** | **Δ** | **t1** | **t2** | **Δ** |
| 2.91 | 2.93 | **N-methylhydantoin** | 3.0 ± 1.1 | 2.4 ± 0.70 | -0.66 ± 0.46^a^ | 3.6 ± 1.4 | 1.9 ± 0.67 | -1.6 ± 0.93^a^ | 3.4 ± 1.2 | 1.9 ± 0.71 | -1.5 ± 0.65 | 3.5 ± 1.1 | 1.7 ± 0.36 | -1.6 ± 1.0 |
| 3.02 | 3.04 | **Creatine** | 16.6 ± 3.8 | 24.5 ± 1.9^b,c^ | 8.4 ± 3.2^b,c^ | 13.7 ± 6.8 | 27.5 ± 20.8^b,c^ | 12.8 ± 22.3^a,c^ | 19.0 ± 2.2 | 77.0 ± 7.8^c,c^ | 57.8 ± 5.9^c,c^ | 15.1 ± 6.2 | 55.7 ± 29.2^b,b^ | 40.4 ± 29.2^a,b^ |
| 3.24 | 3.26 | **Trimethylamine** | 9.3 ± 2.7 | 13.6 ± 6.3 | 4.0 ± 5.1 | 10.5 ± 6.8 | 22.4 ± 18.8 | 11.9 ± 16.1 | 11.4 ± 4.4 | 19.3 ± 16.2 | 8.9 ± 16.3 | 6.5 ± 2.2 | 8.2 ± 4.4 | 1.5 ± 4.1 |
| 3.74 | 3.76 | **Gluconate** | 18.4 ± 1.4 | 16.4 ± 1.1^c,c^ | -2.0 ± 1.5^c,c^ | 18.5 ± 2.5 | 49.6 ± 8.3^a,c,c^ | 31.5 ± 7.1^b,c,c^ | 18.7 ± 1.4 | 41.0 ± 7.4^a,c,c^ | 22.1 ± 7.3^b,c,c^ | 17.6 ± 2.4 | 17.9 ± 4.7^c,c^ | 0.69 ± 5.6^c,c^ |
| 4.02 | 4.05 | **Creatinine** | 17.8 ± 1.6 | 22.1 ± 1.5^a,c,c^ | 4.4 ± 0.96 | 16.9 ± 2.8 | 29.0 ± 3.5^a^ | 11.5 ± 4.5 | 18.6 ± 1.6 | 33.4 ± 2.2^c^ | 14.8 ± 2.6 | 20.7 ± 9.5 | 33.9 ± 10.3^c^ | 12.4 ± 17.4 |
| 4.23 | 4.29 | **Threonine** | 4.6 ± 1.3 | 4.6 ± 0.8 | 0.08 ± 1.4 | 5.3 ± 1.0 | 4.5 ± 1.7 | -0.60 ± 1.9 | 5.4 ± 1.4 | 4.6 ± 1.3 | -0.97 ± 1.5 | 5.5 ± 1.2 | 5.2 ± 1.3 | -0.12 ± 1.1 |
| 5.18 | 5.22 | **N-glucosamine** | 0.97 ± 0.45 | 1.1 ± 0.23 | 0.09 ± 0.55 | 1.2 ± 0.23 | 2.1 ± 5.2 | 0.95 ± 5.2 | 1.1 ± 0.24 | 0.48 ± 0.12 | -0.56 ± 0.27 | 1.1 ± 0.34 | 0.59 ± 0.35 | -0.45 ± 0.37 |
| 5.23 | 5.25 | **Maltose** | 2.9 ± 0.33 | 3.0 ± 0.41 | 0.07 ± 0.58 | 2.5 ± 0.66 | 2.8 ± 2.0 | 0.28 ± 2.0 | 2.8 ± 0.53 | 1.6 ± 0.56 | -1.2 ± 0.71 | 2.7 ± 0.86 | 1.9 ± 0.88 | -0.78 ± 1.3 |
| 5.25 | 5.26 | **Galactose** | 0.52 ± 0.17 | 0.64 ± 0.12 | 0.13 ± 0.16^a^ | 0.57 ± 0.20 | 0.60 ± 0.28 | 0.04 ± 0.30 | 0.68 ± 0.14 | 0.68 ± 0.16 | -0.02 ± 0.19 | 0.70 ± 0.23 | 0.45 ± 0.20 | -0.21 ± 0.24^a^ |
| 5.4 | 5.42 | **Sucrose** | 1.7 ± 0.67 | 2.0 ± 0.59^a,b^ | 0.30 ± 0.60^b^ | 1.4 ± 0.39 | 1.0 ± 0.88^a^ | -0.35 ± 0.87 | 1.7 ± 0.68 | 0.76 ± 0.29^b^ | -1.0 ± 0.66^b^ | 1.7 ± 0.63 | 1.4 ± 0.70 | -0.39 ± 0.75 |
| 5.82 | 5.84 | **Xanthosine** | 3.8 ± 1.5 | 5.9 ± 2.2 | 2.0 ± 2.1 | 2.8 ± 2.0 | 4.7 ± 4.6 | 1.7 ± 4.5 | 3.5 ± 1.6 | 5.6 ± 4.6 | 2.4 ± 4.4 | 3.0 ± 1.3 | 3.2 ± 1.2 | 0.17 ± 1.2 |
| 5.86 | 5.88 | **Cytidine** | 1.7 ± 0.71 | 2.5 ± 0.91 | 0.89 ± 0.96 | 1.2 ± 0.91 | 2.2 ± 2.4 | 0.89 ± 2.4 | 1.6 ± 0.67 | 2.6 ± 2.3 | 1.0 ± 2.0 | 1.4 ± 0.59 | 1.4 ± 0.53 | -0.05 ± 0.72 |
| 5.97 | 6.03 | **Allantoin** | 4.9 ± 1.2 | 6.3 ± 1.9^b^ | 1.5 ± 2.1^b^ | 4.2 ± 1.6 | 4.4 ± 1.8 | -0.03 ± 2.6 | 5.1 ± 0.63 | 4.4 ± 0.98 | -0.78 ± 0.93 | 5.5 ± 1.1 | 3.7 ± 1.5^b^ | -1.7 ± 2.0^b^ |
| 6.23 | 6.27 | **2-deoxyuridine** | 0.50 ± 0.35 | 0.51 ± 0.22 | 0.05 ± 0.45^a^ | 0.51 ± 0.25 | 0.46 ± 0.51 | -0.08 ± 0.55 | 0.63 ± 0.14 | 0.45 ± 0.15 | -0.18 ± 0.19 | 0.78 ± 0.27 | 0.25 ± 0.26 | -0.51 ± 0.17^a^ |
| 6.52 | 6.53 | **Fumarate** | 0.24 ± 0.13 | 0.15 ± 0.12^a,a^ | -0.09 ± 0.13^a^ | 0.20 ± 0.14 | 0.07 ± 0.12 | -0.16 ± 0.18 | 0.28 ± 0.18 | 0.03 ± 0.03^a^ | -0.25 ± 0.20 | 0.35 ± 0.14 | 0.02 ± 0.03^a^ | -0.31 ± 0.15^a^ |
| 6.83 | 6.86 | **3-hydroxyphenylacetate** | 1.5 ± 0.61 | 1.7 ± 0.22^a,c^ | 0.19 ± 0.69^c^ | 1.8 ± 0.34 | 4.7 ± 1.4^c,c,c^ | 2.9 ± 1.3^c,c,c^ | 1.7 ± 0.20 | 2.9 ± 0.73^a,b,c^ | 1.2 ± 0.86^a,c^ | 1.6 ± 0.22 | 1.5 ± 0.37^b,c^ | -0.04 ± 0.29^a,c^ |
| 7.06 | 7.09 | **N-phenylacetylphenylalanine** | 1.9 ± 0.62 | 1.9 ± 0.41^c,c,c^ | -0.03 ± 0.83^b,b,b^ | 2.0 ± 0.42 | 0.83 ± 0.40^c^ | -1.1 ± 0.58^b^ | 2.0 ± 0.45 | 0.81 ± 0.22^c^ | -1.2 ± 0.43^b^ | 1.8 ± 0.49 | 0.49 ± 0.18^c^ | -1.2 ± 0.49^b^ |
| 7.13 | 7.14 | **Indole-3-acetate** | 0.43 ± 0.23 | 0.50 ± 0.12 | 0.06 ± 0.28 | 0.49 ± 0.07 | 0.55 ± 0.18 | 0.08 ± 0.20 | 0.50 ± 0.07 | 0.45 ± 0.24 | -0.03 ± 0.22 | 0.48 ± 0.15 | 0.33 ± 0.16 | -0.14 ± 0.25 |
| 7.22 | 7.28 | **3-indoxylsulfate** | 5.6 ± 1.1 | 5.5 ± 0.70^a,c^ | -0.11 ± 1.5^a,b^ | 6.2 ± 1.3 | 9.5 ± 2.1^c,c^ | 3.4 ± 2.3^b,b^ | 5.7 ± 0.56 | 8.5 ± 3.0^a,c^ | 2.8 ± 3.0^a,b^ | 5.2 ± 1.1 | 4.1 ± 1.5^c,c^ | -0.91 ± 1.7^b,b^ |
| 7.37 | 7.39 | **Phenylalanine** | 1.1 ± 0.35 | 1.1 ± 0.15^c,c^ | 0.03 ± 0.44^c,c^ | 1.2 ± 0.33 | 2.5 ± 0.46^a,c,c^ | 1.3 ± 0.40^c,c^ | 1.2 ± 0.08 | 2.0 ± 0.48^a,c,c^ | 0.86 ± 0.50^c,c^ | 1.1 ± 0.24 | 1.1 ± 0.17^c,c^ | -0.01 ± 0.22^c,c^ |
| 7.44 | 7.5 | **Benzoate** | 4.1 ± 1.2 | 4.0 ± 0.52^c,c^ | -0.13 ± 1.5^c,c^ | 4.6 ± 0.92 | 14.5 ± 3.4^c,c^ | 10.0 ± 3.3^c,c^ | 4.5 ± 0.47 | 11.7 ± 3.7^c,c^ | 7.3 ± 3.9^c,c^ | 3.9 ± 0.96 | 4.7 ± 1.7^c,c^ | 0.87 ± 2.3^c,c^ |
| 7.81 | 7.84 | **Hippurate** | 18.0 ± 2.7 | 16.9 ± 1.6^c,c,c^ | -1.2 ± 2.5^b,c,c^ | 16.7 ± 1.9 | 10.5 ± 1.4^c,c,c^ | -6.2 ± 2.3^b,c,c^ | 17.7 ± 1.7 | 5.5 ± 1.4^c,c,c^ | -12.0 ± 2.1^c,c^ | 16.6 ± 3.7 | 2.3 ± 0.75^c,c,c^ | -13.8 ± 3.8^c,c^ |
| 8.02 | 8.08 | **3-methylxantine** | 2.2 ± 1.1 | 2.4 ± 0.41^b^ | 0.22 ± 1.3 | 2.1 ± 0.62 | 2.0 ± 0.87 | -0.01 ± 0.95 | 2.4 ± 0.24 | 1.8 ± 0.61 | -0.57 ± 0.66 | 2.3 ± 0.57 | 1.4 ± 0.55^b^ | -0.76 ± 0.61 |
| 8.14 | 8.19 | **1-methylnicotinamide** | 0.86 ± 0.56 | 1.2 ± 0.53 | 0.29 ± 0.87 | 1.1 ± 0.37 | 1.6 ± 0.66 | 0.48 ± 0.67 | 1.2 ± 0.30 | 1.4 ± 0.31 | 0.11 ± 0.36 | 1.2 ± 0.34 | 1.2 ± 0.48 | 0.07 ± 0.48 |
| 8.45 | 8.47 | **Formate** | 1.3 ± 0.47 | 0.78 ± 0.25 | -0.47 ± 0.60 | 1.3 ± 0.34 | 0.77 ± 0.50 | -0.47 ± 0.60 | 1.4 ± 0.53 | 0.56 ± 0.20 | -0.82 ± 0.59 | 1.7 ± 0.67 | 0.56 ± 0.56 | -1.0 ± 0.52 |
| 8.81 | 8.85 | **Pyrimidine** | 0.39 ± 0.33 | 0.41 ± 0.15 | 0.02 ± 0.49 | 0.37 ± 0.13 | 0.40 ± 0.26 | 0.05 ± 0.26 | 0.47 ± 0.08 | 0.31 ± 0.15 | -0.16 ± 0.16 | 0.44 ± 0.12 | 0.28 ± 0.22 | -0.15 ± 0.22 |

a = statistical difference between groups at the corresponding column and row (p < 0.05)

b = statistical difference between groups at the corresponding column and row (p < 0.01)

c = statistical difference between groups at the corresponding column and row (p < 0.001)
